# Tests of covariate effects under finite Gaussian mixture regression models

**DOI:** 10.1080/02664763.2024.2433567

**Published:** 2024-11-27

**Authors:** Chong Gan, Jiahua Chen, Zeny Feng

**Affiliations:** a Department of Mathematics and Statistics, University of Guelph, Guelph, Canada; b Department of Statistics, University of British Columbia, Vancouver, Canada

**Keywords:** Cluster analysis, mixture models, hypothesis test, Gaussian mixture regression, Chiroptera data

## Abstract

Mixture of regression model is widely used to cluster subjects from a suspected heterogeneous population due to differential relationships between response and covariates over unobserved subpopulations. In such applications, statistical evidence pertaining to the significance of a hypothesis is important yet missing to substantiate the findings. In this case, one may wish to test hypotheses regarding the effect of a covariate such as its overall significance. If confirmed, a further test of whether its effects are different in different subpopulations might be performed. This paper is motivated by the analysis of Chiroptera dataset, in which, we are interested in knowing how forearm length development of bat species is influenced by precipitation within their habitats and living regions using finite Gaussian mixture regression (GMR) model. Since precipitation may have different effects on the evolutionary development of the forearm across the underlying subpopulations among bat species worldwide, we propose several testing procedures for hypotheses regarding the effect of precipitation on forearm length under finite GMR models. In addition to the real analysis of Chiroptera data, through simulation studies, we examine the performances of these testing procedures on their type I error rate, power, and consequently, the accuracy of clustering analysis.

## 1. Introduction

Bats (Chiroptera) are sensitive to environmental changes, and are often used as indicators for ecosystem changes in their habitats [17]. Studying how the climate influences the forearm structure can provide valuable insights into the evolutionary adaptations of bats in response to different environmental conditions. In particular, we are interested in how the bat's forearm evolved subject to the precipitation impact. This relationship is not yet well understood. Substantial precipitation may cause certain roosts uninhabitable, particularly those in low-lying areas or caves, and therefore such environmental challenges might either force the bats to seek alternative roosts that require broader wings for long-distance flight or adapt their forearms for better movement in moisture-rich conditions [13]. Increased rainfall can increase the insect population and activity. Bats in Microchiroptera, predominantly insectivorous, tend to have shorter wings to facilitate rapid flight and provide greater agility [12]. While rain can also influence the growth of plants, most bats in the Megachiroptera suborder, which feed on fruit, nectar, and flowers, possess longer forearms that facilitate lower flight when approaching fruit trees and allow them to transport heavy fruits back to their roosts [10]. It seems the precipitation might have different effects on the evolutionary development of forearm of different bat species. We acquired trait data of bat species that include the forearm length and the average monthly precipitation in their habitat regions for 839 bat species worldwide from the study of Jones et al. [8]. We aim to determine whether precipitation affects forearm length development differently with respect to different underlying groups of bats. To account for these heterogeneous effects, a finite mixture of regression models can be used. Hereafter, we will use the simpler term ‘mixture’ for ‘finite mixture’.

Under a mixture model, the variable *y* of interest has a distribution with a density function given by

(1) $$f(y;\Theta )=\sum_{g=1}^{G}\pi_{g}f(y;\theta_{g}),$$

where $f(y;\theta_{g})$ is a density function from a parametric family $\left\{f(y;\theta_{g}):\theta_{g}\in \Theta \right\}$, *G* is a positive integer representing the order of the mixture model, $\pi_{1},\ldots ,\pi_{G}$ are non-negative mixing proportions such that $\sum_{g}\pi_{g}=1$. When $f(y;\theta_{g})$ is a normal density for g=1,…,G, we call it a Gaussian mixture.

Mixture model has a latent variable interpretation. One may postulate that there is an unobserved latent vector of indicator variables $Z_{i}=(Z_{i1},\ldots ,Z_{\mathrm{iG}})$ associated with each sample, where $Z_{\mathrm{ig}}$ takes value of 0 or 1 with $Z_{\mathrm{ig}}=1$ indicating the *i*th observation being from the latent *g*th component, or, 0 otherwise, and $\sum_{g=1}^{G}Z_{\mathrm{ig}}=1$. Given $Z_{\mathrm{ig}}=1$, the response *y* has distribution $f(y;\theta_{g})$. Suppose in addition to the indicator variables, each sample is also associated with some covariates/predictors $x=(1,x_{1},\ldots ,x_{p}{)}^{⊤}$ such that the conditional distribution of *y* given $Z_{\mathrm{ig}}=1$ and x has density $f(y|x;\theta_{g})$ characterized by a regression relationship. When a linear regression model is assumed for each subpopulation, we have

(2) $$y|(Z_{\mathrm{ig}}=1)={\text{β}}_{g}^{⊤}x+\epsilon$$

with subpopulation specific regression coefficient

$${\text{β}}_{g}^{⊤}=({\text{β}}_{0g},{\text{β}}_{1g},\ldots ,{\text{β}}_{\mathrm{pg}}).$$

With respect to our motivated analysis of Chiroptera data, where forearm length of a bat species is the response *Y* and precipitation is the only covariate, we focus on one predictor/covariate model with a Gaussian error distribution. In this case *p* = 1, ${\text{β}}_{g}^{T}=({\text{β}}_{0g},{\text{β}}_{1g})$, and a constant term of 1 is add to $x_{i}$ such that ${\text{β}}_{g}^{⊤}x_{i}={\text{β}}_{0g}+{\text{β}}_{1g}x_{i}$. This turns into a Gaussian mixture regression (GMR) model with one covariate.

Mixture regression models have been widely used to explore and model the heterogeneous covariate effects on the response. In customer value analysis, a GMR model, instead of a simple linear regression (SLR) model, was used to relate the sale and the advertising dollar amount because the GMR can capture different relationships across subpopulations. The subpopulations can be formed by declined, mature, and growing industries [5], for instance. In criminal psychology, GMR was used to understand how known factors such as distress, trauma, and personality are associated with drug abuse among juvenile offenders [11]. GMR provides greater specificity and better assists policymakers and practitioners to understand and intervene accordingly to substance-related subgroup differences.

Although mixture regression models are widely used in real applications, testing for covariate effects is often overlooked. The objective of this paper is to develop testing procedures for hypotheses regarding the effects of covariates. Particularly, in the Chiroptera study, where there is only one covariate in the GMR model, we consider the following hypothesis tests:

(3) $$\begin{array}{rl} & \text{(a) Overall effect test:}\;H_{0}:{\text{β}}_{11}={\text{β}}_{12}=\cdots ={\text{β}}_{1G}=0,\\ & \;H_{a}:{\text{β}}_{1g}\neq 0\;\text{for some}\;g; \end{array}$$

(4) $$\begin{array}{rl} & \text{(b) Heterogeneous effect test:}\;H_{0}:{\text{β}}_{11}={\text{β}}_{12}=\cdots ={\text{β}}_{1G},\\ & \;H_{a}:{\text{β}}_{1g}\neq {\text{β}}_{1g^{\prime }}\;\text{for some}\;g\neq g^{\prime }. \end{array}$$

In (a), we aim to determine whether a covariate has significant effects such that this covariate should be included in the mixture regression model. In (b), we aim to determine whether the effects of this covariate differ significantly from one subpopulation to another. To the best of our knowledge, the test problem of this nature has received little to no attention in the literature so far.

In general, scientists cannot unequivocally propose an order for the mixture or mixture regression model. In such applications, one may first employ an order selection procedure. However, this convention leads to a random order *G* that invalidates tests developed for fixed *G* in principle. To mitigate this issue, we design several testing procedures taking random *G* problem into consideration. For proof of concept, our study only considers one covariate under GMR framework. We investigate the properties of these proposed procedures and examine their performances via simulation experiments and real data analyses. In real data analyses, we apply our proposed procedures for the overall test, heterogeneous effect test and sequential test to the Chiroptera data and the diabetes data. Application to Chiroptera data will be presented in Section 5. To demonstrate the broad application of the proposed methods, application to the diabetes data will be in Section 4.2 in the Supplementary Materials.

This paper is organized as follows. In Section 2, we present the proposed hypothesis tests. Our proposed testing procedures are described in Section 3. We present simulation results in Section 4. Application to Chiroptera data is given in Section 5. Section 6 contains summary and discussions.

## 2. Proposed tests

In this paper, we focus on hypothesis tests (a) and (b) as specified in (3) and (4) in the introduction section. These hypotheses can be tested separately or sequentially.

Suppose we have a set of random sample $(y_{i},x_{i}),i=1,\ldots ,n$ from a GMR model. We assume that $x_{i}$'s have a distribution with positive and finite variance that is not related to the parameters of the GMR. Let $\phi (\cdot ;{\text{β}}_{g}^{T}x_{i},\sigma^{2})$ denote a normal density with mean of ${\text{β}}_{g}x_{i}$ and variance of $\sigma^{2}$. The log likelihood function of Θ under GMR can then be written as

(5) $$\ell (\Theta )=\sum_{i=1}^{n}\log\left\{\sum_{g=1}^{G}\pi_{g}\phi (y_{i};{\text{β}}_{g}^{⊤}x_{i},\sigma^{2})\right\}.$$

In the following subsections, we present some likelihood based tests for the overall effect test, heterogeneous effect test, and sequential test.

### 2.1. Test for overall effect

To investigate the appropriateness of a GMR model, one may wish to first determine whether a covariate being considered has any effect on the response at all. This consideration leads to the hypothesis test (a) specified in display (3). When $H_{0}$ holds, the GMR is reduced to an ordinary Gaussian mixture (GM) model where in (5), $\phi (y_{i};{\text{β}}_{0g},\sigma^{2})$ is a normal density that has a mean of ${\text{β}}_{0g}$, which is independent of $x_{i}$, and a variance of $\sigma^{2}$. When the order *G* of GMR is pre-specified, we propose to employ the likelihood ratio test (LRT). More specifically, let ${\hat{\Theta }}_{\mathrm{GM}}$ and ${\hat{\Theta }}_{\mathrm{GMR}}$ be the MLEs of parameters under the null model and the full model for alternative hypothesis as given by (3). The LRT statistic is given by

(6) $$\mathrm{LRT}=-2\{\ell ({\hat{\Theta }}_{\mathrm{GM}})-\ell ({\hat{\Theta }}_{\mathrm{GMR}})\}.$$

The null limiting distribution of LRT statistic in (6) is $\chi_{G}^{2}$, the same as regular models when *G* is known. We use EM algorithm to obtain values of ${\hat{\Theta }}_{\mathrm{GM}}$ and ${\hat{\Theta }}_{\mathrm{GMR}}$ in applications. The stopping criterion we adopted is based on the incomplete-data log-likelihood. When the change in log-likelihood is less than the pre-specified tolerance value $10^{-5}$, the EM algorithm stops. In practice, one should use many initial values for the EM algorithm to lower the risk of local maxima. In our data analysis, we implement this approach.

### 2.2. Test for heterogeneity effect

In some studies, the covariate under investigation is known to affect the response, or its overall effect has been shown to be significant. We may then wonder whether the covariate has the same effect on the response across all subpopulations or it has heterogeneous effects depending on the underlying subpopulation. We can answer this question by testing for the hypotheses (b) as specified in (4). Under $H_{0}$, regression coefficients ${\text{β}}_{1g}$'s are all equal and not zero. We denote the MLEs of Θ under this restriction as ${\hat{\Theta }}_{\mathrm{Homo}-\mathrm{GMR}}$. Note that the intercepts ${\text{β}}_{0g}$'s can vary depending on *g*. Hereafter, we refer to the GMR model under such restriction as the Homo-GMR model. The $H_{a}$ in (4) simply corresponds to the unrestricted GMR that allows ${\text{β}}_{1g}$'s to be different. The LRT statistic for hypothesis test in (4) is given by

(7) $$\mathrm{LRT}=-2(\ell ({\hat{\Theta }}_{\mathrm{Homo}-\mathrm{GMR}})-\ell ({\hat{\Theta }}_{\mathrm{GMR}})).$$

When *G* is fixed and known, this LRT statistic has an asymptotic $\chi_{G-1}^{2}$ distribution. As there is no solution for finding the MLEs of $\Theta_{\mathrm{Homo}-\mathrm{GMR}}$ in literature, we present our derived EM algorithm for finding the restricted MLEs, ${\hat{\Theta }}_{\mathrm{Homo}-\mathrm{GMR}}$, in Section 1 in the Supplementary Materials.

### 2.3. Sequential test

In applications, one may first test for the overall effect to assert the necessity of a GMR model as opposed to a more parsimonious GM model. After a significant outcome of the test, one may in addition question the necessity of GMR or a Homo-GMR model as in $H_{0}$ in (4) suffices or is more suitable. This consideration leads to the notion of sequential tests. If the overall effect is judged not significant, one accepts the parsimonious GM model. Otherwise, one further tests the heterogeneity of covariate effects to choose between a Homo-GMR or a GMR model.

## 3. Significance tests when order is unknown

The likelihood ratio test requires a known order of the mixture models under $H_{0}$ and $H_{a}$ to specify the degrees of freedom for the $\chi^{2}$ distribution. However, the order is usually not known in applications and is typically selected among candidate models based on some selection criteria. The inferred order naturally inherits the uncertainty of data and leads to technical challenges to the validity of a hypothesis test. To mitigate this difficulty, we propose three Naive test procedures and a weighted significance test (WEST) procedure for the hypothesis tests (a) and (b) when the order is unknown.

We now review the Bayesian information criterion (BIC) of Schwarz [16]. Let the competing candidate models be $M_{1},\ldots ,M_{J}$, and $\Theta_{j}$ be the parameter vector of model $M_{j}$. Suppose we have a dataset of size *n* and it leads to *J* log-likelihood functions, $\ell_{j}(\Theta_{j})$, for models $M_{1},\ldots ,M_{J}$ respectively. Let ${\hat{\Theta }}_{j}$ be the MLEs of $\Theta_{j}$ under the model $M_{j}$ and $p_{j}$ be the dimension of the parameter vector of $\Theta_{j}$. The classical BIC is then defined to be

(8) $$\text{BIC}(M_{j})=-2\ell_{j}({\hat{\Theta }}_{j})+p_{j}\log n.$$

When these models are regular, $\text{BIC}(M_{j})$ approximates the negative log posterior probability of model $M_{j}$ given some prior probabilities. Hence, an optimal choice among these candidate models is often taken as the one that has the minimum BIC value.

When candidate models are not regular, as in the case of mixture regression, $\text{BIC}(M_{j})$ is not optimal but remains a good criterion because it balances the desire of having a good fit and the lower model complexity. While there are many other criteria developed in the context of mixture and mixture regression, none of them have gained universal endorsement. Thus the classical BIC remains a popular choice. We adopt the BIC for order selection in this article with one important alternation. Note that each model $M_{j}$ has a fixed order but has two versions: one corresponds to $H_{0}$ (GM or Homo-GMR) and the other corresponds to $H_{a}$ (GMR). The BIC ( $M_{j}$) requires specification of either under $H_{0}$ or $H_{a}$. On the basis of BIC outcomes, we create several testing procedures for the significance of covariate effects and the optimal model for clustering will be determined as well.

### 3.1. Procedures for overall and heterogeneous effects tests

Given a set of candidate orders {2,…,J} that is for both under $H_{0}$ and $H_{a}$, we propose the following testing procedures for the significance of covariate effects that apply to both hypothesis tests (a) and (b) as specified in (3) and (4). Here *J* is a positive integer greater than 2.

**Naive I procedure:**

Step 1: Select the order *G* among {2,…,J} that has the smallest BIC value among all fitted models under $H_{0}$ and $H_{a}$. Let $M_{G}$ denote models of order *G* under $H_{0}$ and $H_{a}$.
Step 2: Compute the observed LRT statistic using (6) or (7) depending on the hypothesis test, denoted as ${\mathrm{LRT}}_{\mathrm{obs}}$, under $M_{G}$ for the corresponding $H_{0}$ and $H_{a}$. At *α* significance level, reject $H_{0}$ if ${\mathrm{LRT}}_{\mathrm{obs}}>\chi_{\mathrm{df},\alpha }^{2}$, or otherwise do not reject the $H_{0}$. The df is the difference of the number of parameters of $M_{G}$ under $H_{a}$ and $H_{0}$.

If $H_{0}$ is rejected, a *G*-component GMR under $H_{a}$ is selected as the preferred model. If $H_{0}$ is not rejected, a *G*-component GM or Homo-GMR is selected as the preferred model depending on which one of the (a) and (b) is being tested.

**Naive II procedure:**

Step 1: Select the order *G* among {2,…,J} that has the smallest BIC value among all fitted models under $H_{0}$. Let $M_{G}$ denote models of order *G* under $H_{0}$ and $H_{a}$.
Step 2: With the selected order *G*, compute ${\mathrm{LRT}}_{\mathrm{obs}}$ under $M_{G}$. Reject $H_{0}$ if ${\mathrm{LRT}}_{\mathrm{obs}}>\chi_{\mathrm{df},\alpha }^{2}$, or otherwise do not reject the $H_{0}$. The df is the difference of the number of parameters of $M_{G}$ under $H_{a}$ and $H_{0}$.

If $H_{0}$ is rejected, a *G*-component GMR is selected as the preferred model. If $H_{0}$ is not rejected, a *G*-component GM or Homo-GMR is selected as the preferred model depending on which one of (a) and (b) is being tested.

**Naive III procedure:**

Step 1: Let $M_{j}$ denote models of order *j* under $H_{0}$ and $H_{a}$ of the test. For each possible order *j* in {2,…,J}, perform the LRT under $M_{j}$. Retain $H_{0}$ version of $M_{j}$ if it is not rejected. Otherwise, retain the $H_{a}$ version of $M_{j}$.
Step 2: Compute BIC values of $M_{j}$ of its $H_{0}$ or $H_{a}$ version decided in Step 1, select the order *G* that has the minimum BIC value. If the selected $M_{G}$ is under $H_{a}$, reject $H_{0}$, or otherwise do not reject the $H_{0}$.

The selected $M_{G}$ model in Step 2 is the preferred model.

Naive I procedure utilizes an intuitive 2-step procedure where the first step is to select an optimal model among orders of {2,…,J} under $H_{0}$ and $H_{a}$, followed by the hypothesis test with the selected order. Naive II procedure is similar to the Naive I except that in the first step, the order is selected among {2,…,J} under $H_{0}$ only. Naive III simply reverses steps of order selection and hypothesis test.

The three Naive procedures are somewhat ad hoc and so, we develop a weighted significance test (WEST) procedure, in which, the evidence of significance of covariate effects is aggregated from all candidate models, $M_{j}$'s. Recall that the BIC value has a posterior probability interpretation. This allows us to assign weights to models according to their BIC values. Let BIC( $M_{j}$) be the BIC value of model $M_{j}$ under $H_{0}$ and let

(9) $$\Pi_{j}=\frac{\exp\left(-\frac{1}{2}\text{BIC}(M_{j})\right)}{\sum_{\text{all}\;j}\exp\left(-\frac{1}{2}\text{BIC}(M_{j})\right)}.$$

Let $T_{j}$ and $t_{j}$ be, respectively, the test statistic and its observed value under model $M_{j}$ of the test. We define a weighted significance level to be

(10) $$p_{0}=\sum_{\text{all}\;j}\Pi_{j}P(T_{j}\geq t_{j}|H_{0},M_{j})=\sum_{\text{all}\;j}\Pi_{j}({p-\mathrm{val}}_{j}).$$

Note that ${\text{p-val}}_{j}=P(T_{j}\geq t_{j}|H_{0},M_{j})$ represents the p-value of the test under model $M_{j}$. We present the WEST procedure below and provide more details about our derived weighted significance level $p_{0}$ in Section 2 in the Supplementary Materials.

**WEST procedure**

Step 1: For each $M_{j}$, fit models under $H_{0}$ and $H_{a}$, compute ${\mathrm{LRT}}_{\mathrm{obs}}$ and the BIC( $M_{j}$) values under $H_{0}$.
Step 2: For each $M_{j}$, compute the ${\text{p-val}}_{j}$ according to
$${p-\mathrm{val}}_{j}=P({\text{LRT}}_{j}\geq {\text{LRT}}_{\mathrm{ob}s_{j}}|H_{0},M_{j})$$
  where ${\text{LRT}}_{j}$ follows a $\chi_{{\mathrm{df}}_{j}}^{2}$ distribution asymptotically with ${\mathrm{df}}_{j}$ being the difference of the number of parameters between $H_{a}$ and $H_{0}$ under $M_{j}$.
Step 3: Use the p-val_*j*_'s and BIC( $M_{j}$) values acquired in Steps 1 and 2 to compute the weighted significance level $p_{0}$ using (10).
Step 4: Reject the $H_{0}$ if $p_{0}$ is smaller than nominal level *α*, or otherwise do not reject $H_{0}$.

If $H_{0}$ is rejected, the preferred model will be under $H_{a}$ version with the order having the minimum BIC value. Otherwise, the preferred model will be under $H_{0}$ version with the order having the minimum BIC value.

### 3.2. Procedures for sequential tests

This section presents procedures for sequential tests as described in Section 2.3.

**Naive procedures**

Step 1: Carry out one of the three Naive procedures (Naive I, II, and III) of your choice for the overall test. If the $H_{0}$ is not rejected, we conclude that the covariate has no significant effect on the response and the procedure is completed. Otherwise, proceed to Step 2.

As a result of Step 1, an order *G* is selected. If the $H_{0}$ is not rejected, a *G*-component GM model is selected.

Step 2: With the selected *G* in Step 1, fit both the Homo-GMR and GMR models.
Step 3: Compute the ${\mathrm{LRT}}_{\mathrm{obs}}$ for the heterogeneous effect test. Reject $H_{0}$ if ${\mathrm{LRT}}_{\mathrm{obs}}>\chi_{G-1,\alpha }^{2}$, or otherwise, do not reject $H_{0}$.

If $H_{0}$ is rejected, there is significant evidence to suggest that the covariate has heterogeneous effects on the response and a *G*-component GMR model is preferred. Otherwise, there is insufficient evidence to suggest that the covariate has heterogeneous effects and a *G*-component Home-GMR is preferred.

**WEST procedure**

Step 1: Perform the overall test using WEST procedure. If the $H_{0}$ is not rejected, we conclude that the covariate has no significant effect on the response and the procedure is completed. Otherwise, proceed to Step 2.

If the $H_{0}$ is not rejected, an order *G* will be selected among all fitted GM models that has the minimum BIC value, the *G*-component GM model is the preferred model.

Step 2: Perform WEST procedure to test for the heterogeneous effects. Reject $H_{0}$ if $p_{0}<\alpha$, otherwise, do nor reject $H_{0}$.

If the $H_{0}$ is rejected, we conclude that the covariate has significant heterogeneous effects on the response. The order *G* will be selected among all fitted GMR models that has the minimum BIC value, and a *G*-component GMR model is preferred. Otherwise, the order *G* will be selected among all fitted Homo-GMR models that has the minimum BIC value, and a *G*-component Homo-GMR model is preferred.

## 4. Simulation study

The simulation study is conducted to assess performances of our proposed procedures for overall test, heterogeneous effect test, and sequential tests. Our simulation study will focus on the performance in two aspects: 1) control of type I error rate and power of the test; and 2) accuracy of the clustering analysis.

### 4.1. Simulation models

#### 4.1.1. Simulation models for the overall test

The simulated data in this section is generated using a 3-component GMR model, with the parameters of π's, β's, and σ's specified according to the estimated values of the fitted model to the diabetes data set, see Table S20 in Supplementary Materials. The membership indicator vector Z is generated from multinomial ( 1,π). The covariate $x_{i}$ is generated by Gamma(2.57, 0.01) distribution as this distribution fits the covariate, steady state plasma glucose (sspg) levels, in the diabetes data well. The response variable $y_{i}$ is then generated from Normal( ${\text{β}}_{0g}+{\text{β}}_{1g}x_{i},\sigma^{2}$) if the subject *i* has $Z_{\mathrm{ig}}=1$. The datasets with $\left(x_{i},y_{i}\right)$ for i=1,…,n are used for the power assessment of the proposed overall hypothesis test procedures.

We generate datasets for type I error assessment by setting ${\text{β}}_{1g}=0$ for all *g* to let $y_{i}$ only depend on $Z_{i}$ but not on $x_{i}$ (see Table 1). In Scenario 1, the parameter values, excluding ${\text{β}}_{1g}=0$, are obtained from the 3-component GMR model fitted to the diabetes data. In Scenarios 2 and 3, we decrease the separation between the three clusters by increasing ${\text{β}}_{02}$ and *σ*, respectively. However, to mimic the reality that the response is likely to be affected by at least one variable which is often not observed as a covariate, we consider a second way to generate the data under $H_{0}$ of overall test. That is, the response variable *y* generated for power assessment is retained in the data, and an independent random variable $x^{*}$ generated from Gamma(2.57, 0.01) distribution that has no effect on *y* will be tested for its effects on the *y* instead. The datasets of $\left(x_{i}^{*},y_{i}\right)$ for i=1,…,n are used for assessing the type I error control. Under the second simulation model, response *y* is generated with an underlying unobserved clustering structure and affected by an unobserved covariate *x*.

**Table 1.** True values of parameters in simulation study to assess the type I error rate for overall test.

Overall test under $H_{0}$					
Scenario	*π*	${\text{β}}_{1}$	${\text{β}}_{2}$	${\text{β}}_{3}$	*σ*
1	(0.15, 0.1, 0.75)	(1549, 0)	(987, 0)	(332, 0)	75
2	(0.15, 0.1, 0.75)	(1549, 0)	(1250, 0)	(332, 0)	75
3	(0.15, 0.1, 0.75)	(1549, 0)	(987, 0)	(332, 0)	150

We consider three scenarios to evaluate the power of four testing procedures by specifying the parameters of β's and *σ* differently, as outlined in Table 2. In Scenario 1, β's and *σ* are specified as the parameters using 3-component GMR model to fit the diabetes data set. In Scenarios 2 and 3, we reduce the effects of the covariate on the response by adjusting coefficients β at different levels, and modify the *σ* value to increase the overlaps among clusters.

**Table 2.** True values of parameters in simulation study to assess the type I error rate and power for overall test.

Parameter settings for the overall test					
Scenario	*π*	${\text{β}}_{1}$	${\text{β}}_{2}$	${\text{β}}_{3}$	*σ*
1	(0.15, 0.1, 0.75)	(1549, −3.82)	(987, −1.31)	(332, 0.30)	75
2	(0.15, 0.1, 0.75)	(1549, −0.382)	(987, −0.131)	(332, 0.03)	75
3	(0.15, 0.1, 0.75)	(1549, −0.764)	(987, −0.262)	(332, 0.06)	300

**Table 3.** True values of parameters in simulation study for heterogeneous effect test.

Parameter settings under $H_{0}$ of homogeneous covariate effects					
Scenario	*π*	${\text{β}}_{1}$	${\text{β}}_{2}$	${\text{β}}_{3}$	*σ*
1	(0.5, 0.3, 0.2)	(5, 1)	(1, 1)	(−3, 1)	1
2	(0.5, 0.3, 0.2)	(3, 1)	(0, 1)	(−3, 1)	1
3	(0.5, 0.3, 0.2)	(3, 1)	(0, 1)	(−3, 1)	$\sqrt{2}$
Parameter settings under $H_{a}$ of heterogeneous covariate effects					
Scenario	*π*	${\text{β}}_{1}$	${\text{β}}_{2}$	${\text{β}}_{3}$	*σ*
1	(0.5, 0.3, 0.2)	(5, 1.6)	(1, 1.5)	(−3, 1)	1
2	(0.5, 0.3, 0.2)	(3, 1.2)	(0, 1.1)	(−3, 1)	1
3	(0.5, 0.3, 0.2)	(3, 1.6)	(0, 1.5)	(−3, 1)	$\sqrt{2}$

For each scenario, we generate 1000 data sets for sample sizes of 500 and 300 respectively. Each simulated data set will be fitted by GM and GMR models with G=2,…,5, respectively and the overall test will be conducted. To evaluate the clustering analysis performance on the selected model according to the hypothesis test result, we compute the percentage of correctly selected models and the mean adjusted rand index (ARI) [6] to measure the accuracy of model selection and membership assignment of each subject in the sample over 1000 replicates generated for each combination of settings.

#### 4.1.2. Simulation models for the heterogeneous effect test

Here, we assume the covariate $x_{i}$ is generated from the standard normal distribution, and response $y_{i}$ follows a 3-component GMR model. When datasets are simulated under $H_{0}$, covariate $x_{i}$ has the same effect on the mean response for all subpopulations. We let ${\text{β}}_{1g}=1$ for all g=1,…,G for type I error rate assessment. Scenario 1 emulates a case where the three subpopulations are well separated. In Scenarios 2 and 3, intercepts ( ${\text{β}}_{0g}$) and variance are changed so that we can see how the proposed test procedures work when the data set is not well separated. To assess the power, datasets are simulated under $H_{a}$ that ${\text{β}}_{1g}$ are set differently for each component *g*. Similarly, for each scenario, we manipulate β and *σ*'s to adjust the effects of the covariate and the spread of the data. Again, 1000 replicates for each sample size of 500 and 300 will be generated for each scenario. Each simulated data set will be fitted by Homo-GMR and GMR models with G=2,…,5 and the heterogeneous effect test will be conducted using the four test procedures.

#### 4.1.3. Simulation models for the sequential test

In the first step of the sequential test, the overall test is conducted. As the simulation study for the type I error assessment has been conducted in Section 4.1.1 for the overall test, it will not repeated here. So, in this section, we only simulate data under two scenarios: 1) the covariate has the same effect on the response regardless of which subpopulation the subject is from, and 2) the covariate has different effects on the response depending on which subpopulation that the subject is from.

Table 4 shows the settings in the sequential test. Additional simulation models and results related to the sequential test are provided in Section 3.1 of the Supplementary Materials. Here, Scenario 1 is used to assess the power of both overall test and type I error rate of the heterogeneous effect test, and Scenario 2 is used to assess the power of overall test and heterogeneous effect test. The values of ${\text{β}}_{0g}$, ${\text{β}}_{1g}$ and *σ* are modified to evaluate the performance of our proposed test procedures under different scenarios.

**Table 4.** True values of parameters in simulation study for sequential test.

Scenario	*π*	${\text{β}}_{1}$	${\text{β}}_{2}$	${\text{β}}_{3}$	*σ*
1	(0.3, 0.5, 0.2)	(5, 0.3)	(1, 0.3)	(−3, 0.3)	1
2	(0.3, 0.5, 0.2)	(5, 0)	(1, 0.2)	(−3, 0.5)	1

For each scenario, we generate 1000 data sets for sample sizes of 500 and 300 respectively. GM, GMR and Homo-GMR models were fitted to each simulated data set and *G* is set to be from 2 to 5. The overall test is first conducted. Then, only the data sets that are rejected by the overall test will be continued to test for the heterogeneity of covariate effects. Finally, the percentage of correctly selected models and the adjusted rand index (ARI) will be measured under each scenario.

### 4.2. Results of simulation study

#### 4.2.1. Results for the overall test

For assessing the type I error control, the datasets were first generated under the null hypothesis of overall test (Table 1), where *Y* was generated under a 3-component GM model without any covariate effect. Four proposed testing procedures for the overall effect were performed and the results are summarized in Table 5. The empirical rejection rates are generally close to the nominal 0.05 significance level. The empirical rejection rates slightly increase as the within group variance for generating the response *Y* increases or sample size decreases. Four testing procedures show the same performance based on the percentage of correctly selected models and mean ARI values.

**Table 5.** Type I error rate of overall test at α=0.05, percentage of correctly selected models, and mean of adjusted rand indices. Datasets were simulated under $H_{0}$ that *x* has no effect on the responses *y* (Table 1). The Naive I, II, III and WEST procedures were performed.

		n=500				n=300			
Scenario		Naive I	Naive II	Naive III	WEST	Naive I	Naive II	Naive III	WEST
1	Type I error rate	0.042	0.042	0.041	0.042	0.047	0.047	0.047	0.047
	% of correct models	0.958	0.958	0.958	0.958	0.953	0.953	0.953	0.953
	Mean ARI	0.999	0.999	0.999	0.999	1.000	1.000	1.000	1.000
2	Type I error rate	0.050	0.050	0.049	0.050	0.059	0.059	0.057	0.058
	% of correct models	0.946	0.946	0.946	0.946	0.940	0.940	0.941	0.940
	Mean ARI	0.993	0.993	0.993	0.993	0.994	0.994	0.994	0.994
3	Type I error rate	0.067	0.067	0.063	0.066	0.067	0.067	0.061	0.063
	% of correct models	0.931	0.931	0.932	0.931	0.926	0.926	0.927	0.927
	Mean ARI	0.965	0.965	0.965	0.965	0.962	0.962	0.962	0.962

Recall that in the second approach for assessing type I error control, the response variable $y_{i}$ is generated under a 3-component GMR model with $y_{i}∼\text{Normal}({\text{β}}_{0g}+{\text{β}}_{1g}x_{i},\sigma^{2})$ for *g* = 1, 2, and 3, and $x_{i}$ is not observed. Datasets $\left(x_{i}^{*},y_{i}\right)$ for i=1,…,n were fitted by the GM and GMR models. The hidden GMR structure introduces dispersion to the marginal distribution of the response within each cluster. The results of the empirical null rejection rates, the number of correctly selected model (3-component GM model), and the mean of adjusted rand indices over 1000 replicates are reported in Table 6. When the effective size ( ${\text{β}}_{1g}$) of the influencing covariate *x* is large as in Scenario 1, using Naive I and III procedures to test the association between *y* and the irrelevant covariate $x^{*}$ results in an inflated type I error rate in general. Using WEST procedure, the empirical type I error rate is relatively closer to the nominal 5% significance level. Additionally, when sample size is large, the type I error rates are much reduced. Following the results of WEST procedure, more corrected models are selected. In terms of the accuracy of membership assignment, the Naive I and III outperform both WEST and Naive II methods. Reducing the effective size of the influencing covariate $x_{i}$ on the $y_{i}$, and testing the effect of irrelevant variable $x_{i}^{*}$ on $y_{i}$, as in Scenarios 2 and 3, the type I error rates are reduced overall. The Naive I and II procedures result in a higher type I error rates than the other procedures. Overall, WEST performs better in controlling the type I error rate and selecting more corrected models, while remaining competitive with other methods in cluster membership assignments, and four procedures show improved performance when sample size is large.

**Table 6.** Type I error rate of overall test at α=0.05, percentage of correctly selected models, and mean of adjusted rand indices. Datasets were simulated under $H_{0}$ that $x^{*}$ has no effect on the responses *y* (Table 2). The Naive I, II, III and WEST procedures were performed.

		n=500				n=300			
Scenario		Naive I	Naive II	Naive III	WEST	Naive I	Naive II	Naive III	WEST
1	Type I error rate	0.239	0.061	0.213	0.052	0.378	0.152	0.361	0.140
	% of correct models	0.120	0.140	0.120	0.153	0.071	0.086	0.077	0.090
	Mean ARI	0.175	0.072	0.174	0.104	0.197	0.083	0.196	0.100
2	Type I error rate	0.077	0.077	0.055	0.065	0.069	0.069	0.058	0.068
	% of correct models	0.792	0.792	0.795	0.801	0.849	0.849	0.849	0.849
	Mean ARI	0.996	0.996	0.996	0.996	0.997	0.997	0.997	0.997
3	Type I error rate	0.057	0.057	0.056	0.055	0.067	0.067	0.067	0.066
	% of correct models	0.008	0.008	0.009	0.009	0.003	0.003	0.003	0.003
	Mean ARI	0.643	0.643	0.643	0.643	0.641	0.641	0.641	0.641

Results of type I error assessment at 1% significance level are shown in Tables S4 – S9 in the Supplementary Materials. A similar pattern of the empirical null rejection rates is observed as seen in the results at 5% significance level. However, more correctly selected models are observed when the significance level is more stringent. This makes sense as the test is more stringent such that it is favored to select the true null 3-component GM model.

**Figure 1.**
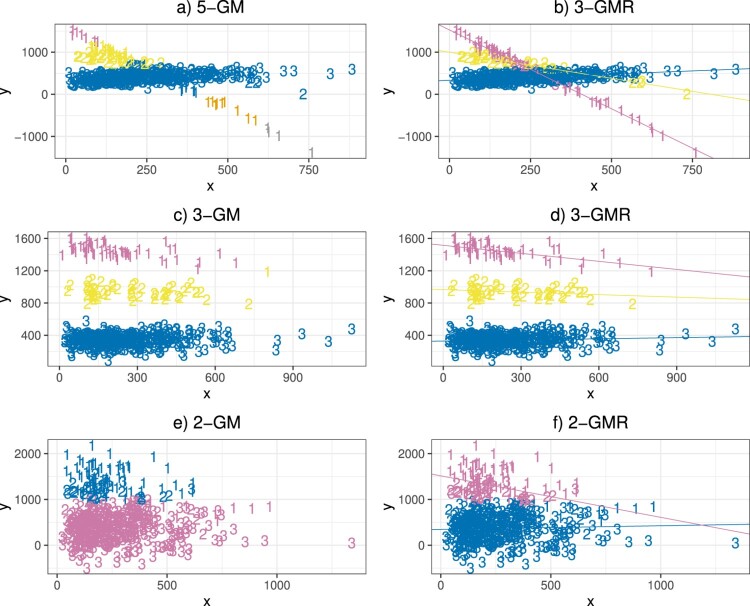
Examples of scatterplots of the best model fitting results to the simulated dataset using GM and GMR models with the optimal order indicated in the title of each plot. The 3 datasets were simulated under $H_{a}$ specified in Table 2, where a) and b) are simulated using parameters for Scenario 1, c) and d) are for Scenario 2, and e) and f) are for Scenario 3. In each plot, the number represents the true membership of each observation, and the color represents the assigned membership by the fitted model.

For the power assessment, datasets of $\left(x_{i},y_{i}\right)$ for i=1,…,n were fitted by the GM and GMR models. The four proposed testing procedures for the overall test were performed. Figure 1 shows examples of data sets of sample size of 500 simulated under three scenarios, where numbers refer to their true memberships and colors refer to the assigned memberships by the fitted model. The number of components is determined according to the lowest BIC among all choices of the orders. For example, for a data set simulated in Scenario 1, 5-GM is the best fitted model among all GM models, and 3-GMR is the best fitted model among all GMR models. Table 7 shows the results of the power, the percentage of correctly selected model (the 3-component GMR model) and the mean of ARI over 1000 replicates. We observe that the power of overall test using Naive and WEST procedures are similar. However, recall that the type I error rates are generally inflated using the Naive I and III methods when the data are generated by the simulation model as specified in Scenario 1. When the covariate *x* has strong effects on responses *y*, following the Naive I, III and WEST procedures, the correct model is more likely to be picked, whereas the Naive II procedure selected less corrected models. This might be because in the Naive II procedure, the first step is to select the order among all fitted models under the $H_{0}$, which would be in favor of GM model without considering the covariate effects, and therefore the Naive II procedure is more likely to pick the incorrect model. The performance of membership assignments (means of the ARI) is similar among all procedures.

#### 4.2.2. Results for the heterogeneous effect test

Table 8 shows the type I error rate, percentage of correctly selected models and mean ARI values for four proposed testing procedures, when performing the heterogeneous effect test at the significance level of 0.05. The data sets are generated under $H_{0}$ that the covariate has the same effect on *y* in all subpopulations. Comparing among all testing procedures, the empirical null rejection rates of WEST procedure are closer to the 0.05 nominal level under all scenarios and sample sizes. The percentage of correctly selected models and the mean adjusted rand index are similar. When sample size is large (n=500), the empirical null rejection rates for all testing procedures are lower than the 0.05 nominal level in Scenario 3, and the Naive III and WEST procedures are even more conservative than the Naive I and II procedures. The percentage of correct models selected and the mean adjusted rand index are similar in general. It is noted that in Scenario 3, when the within-cluster standard deviation is larger, the simulated responses spread wider and would be harder for clustering. So, the percentage of correctly selected models is generally lower for Scenario 3. Overall, the WEST procedure outperforms the others.

**Table 7.** Power of overall test at α=0.05, percentage of correctly selected models, and mean of adjusted rand indices. Datasets were simulated under $H_{a}$ that *x* has effects on the responses *y* (Table 2). The Naive I, II, III and WEST procedures were performed.

		n=500				n=300			
Scenario		Naive I	Naive II	Naive III	WEST	Naive I	Naive II	Naive III	WEST
1	Power	1	1	1	1	1	1	1	1
	% of correct models	0.877	0.338	0.877	0.877	0.794	0.159	0.794	0.794
	Mean ARI	0.772	0.726	0.772	0.772	0.764	0.710	0.764	0.764
2	Power	1	1	0.999	1	0.987	0.986	0.986	0.987
	% of correct models	0.999	0.863	0.999	0.999	0.985	0.902	0.985	0.985
	Mean ARI	1.000	0.999	1.000	1.000	1.000	0.999	1.000	1.000
3	Power	0.680	0.680	0.671	0.676	0.410	0.410	0.408	0.408
	% of correct models	0.006	0.008	0.001	0.001	0.000	0.000	0.000	0.000
	Mean ARI	0.649	0.649	0.649	0.650	0.644	0.644	0.644	0.644

**Table 8.** Type I error rate of heterogeneous effect test at α=0.05, percentage of correctly selected models, and mean of adjusted rand indices. Datasets were simulated under $H_{0}$ that *x* has homogeneous effect on the responses *y* (Table 3). The Naive I, II, III and WEST procedures were performed.

		n=500				n=300			
Scenario		Naive I	Naive II	Naive III	WEST	Naive I	Naive II	Naive III	WEST
1	Type I error rate	0.052	0.052	0.052	0.052	0.067	0.067	0.066	0.066
	% of correct models	0.945	0.945	0.945	0.945	0.931	0.931	0.931	0.931
	Mean ARI	0.916	0.916	0.916	0.916	0.916	0.916	0.916	0.916
2	Type I error rate	0.070	0.069	0.069	0.068	0.061	0.061	0.053	0.051
	% of correct models	0.925	0.926	0.926	0.926	0.864	0.864	0.865	0.873
	Mean ARI	0.768	0.768	0.768	0.768	0.746	0.746	0.744	0.745
3	Type I error rate	0.047	0.047	0.042	0.042	0.058	0.058	0.057	0.050
	% of correct models	0.079	0.079	0.081	0.081	0.059	0.059	0.059	0.061
	Mean ARI	0.459	0.459	0.459	0.459	0.456	0.456	0.455	0.456

The results of power analysis for heterogeneous effect test are summarized in Table 9. The results are similar among Naive and WEST procedures for well-separate datasets (Scenario 1). When the observations from different components are overlapped (Scenarios 2 and 3), we find that a slightly higher power is obtained by using the Naive I and II procedures, but in those scenarios, the type I error rates are slightly higher using Naive methods. Percentage of correctly selected models and mean ARI values are similar in all four testing procedures. It is noted that in Scenarios 2 and 3, simulated data are less well separated, it would be harder for clustering, which leads to a lower percentage of correctly selected models.

**Table 9.** Power of heterogeneous effect test at α=0.05, percentage of correctly selected models, and mean of adjusted rand indices. Datasets were simulated under $H_{a}$ that *x* has heterogeneous effect on the responses *y* (Table 3). The Naive I, II, III and WEST procedures were performed.

		n=500				n=300			
Scenario		Naive I	Naive II	Naive III	WEST	Naive I	Naive II	Naive III	WEST
1	Power	0.990	0.990	0.990	0.990	0.891	0.885	0.889	0.889
	% of correct models	0.989	0.977	0.989	0.989	0.890	0.890	0.888	0.888
	Mean ARI	0.913	0.913	0.913	0.913	0.912	0.911	0.911	0.911
2	Power	0.237	0.237	0.233	0.235	0.171	0.170	0.155	0.152
	% of correct models	0.235	0.236	0.229	0.230	0.161	0.164	0.141	0.139
	Mean ARI	0.766	0.766	0.764	0.765	0.742	0.743	0.737	0.741
3	Power	0.721	0.721	0.716	0.712	0.538	0.537	0.531	0.524
	% of correct models	0.056	0.073	0.044	0.046	0.024	0.027	0.014	0.014
	Mean ARI	0.449	0.451	0.448	0.448	0.446	0.446	0.445	0.445

#### 4.2.3. Results for the sequential test

For sequential test, Table 10 summarizes the sequential test results for the data generated under 3-component Homo-GMR models and under 3-component GMR models. The overall test is first performed. The power of the overall test for each scenario is listed in Tables 10. If the overall test is rejected, heterogeneous effect test is performed. In this case, when calculating the rejection rate for the heterogeneous effect test, only those cases whose null hypothesis in the overall test is rejected will be included. Therefore, the number of heterogeneous effects tests depends on the power of the overall test. The percentage of correctly selected models and the mean ARI values are also reported in the table. When data is generated under 3-component Homo-GMR model, which corresponds to the null hypothesis of heterogeneous effect test, the empirical null rejection rate in the heterogeneous effect tests is close to the nominal level (see Scenario 1). Among four procedures, WEST performs better in general. When the data is generated under the 3-component GMR model, which corresponds to the alternative hypothesis of the heterogeneous effect test, the empirical null rejection rate is the power of the heterogeneous effect test. Four procedures perform similarly.

**Table 10.** The results of sequential test at α=0.05.The Naive I, II, III and WEST procedures were performed.

		n=500				n=300			
Scenario		Naive I	Naive II	Naive III	WEST	Naive I	Naive II	Naive III	WEST
1	Power	0.991	0.991	0.990	0.991	0.932	0.932	0.927	0.932
	(Overall test)								
	Type I error rate	0.054	0.054	0.055	0.054	0.060	0.060	0.060	0.059
	(Heterogeneous test)								
	% of correct models	0.935	0.936	0.934	0.934	0.869	0.869	0.865	0.935
	Mean ARI	0.881	0.881	0.881	0.881	0.897	0.897	0.897	0.897
2	Power	0.977	0.977	0.970	0.978	0.851	0.851	0.836	0.849
	(Overall test)								
	Power	0.882	0.882	0.887	0.883	0.620	0.619	0.620	0.631
	(Heterogeneous test)								
	% of correct models	0.862	0.858	0.860	0.864	0.619	0.613	0.612	0.631
	Mean ARI	0.895	0.895	0.895	0.895	0.893	0.893	0.893	0.893

The percentage of correctly selected models and the mean ARI values are all similar among four testing procedures under all scenarios. All procedures perform better when sample size is larger.

## 5. Application to chiroptera data

### 5.1. Data description

PanTHERIA [8] is a database that offers an exhaustive compilation of life-history, ecological and geographical information for all known mammalian species. It aggregates multispecies trait data from 3143 literature sources, including journals such as Mammalian Species, Journal of Mammalogy and Mammlia, as well as other mammal databases such as Bat Database [7] and Carnivore Database [2]. Some environmental and anthropogenic factors were also collected in the database. Finalized in June 2004, PanTHERIA is accessible through Ecological Archives [8] and stands as an invaluable asset for macroecological and macroevolutionary studies on mammals. We extracted species of Chiroptera order and constructed a new dataset consisting 839 samples in two variables: forearm length and precipitation. The adult forearm length (mm) measures the distance between the elbow and wrist, which is a proxy for understanding the evolutionary adaptations of bats, while precipitation (mm) denotes average monthly rainfall surrounding the bats' habitat and activity regions, which is an environmental variable that might have an effect on the bat over evolutionary time and one of the climate types the bats adapted to. In addition, we included the variable of trophic level for each species, categorizing them as herbivore, carnivore, or omnivore. Salinas-Ramos et al. reported the dietary patterns of the different bat species are driven by prey availability [15]. The trophic level of bats can differ between different habitats or regions, and it can shift throughout the season [14]. Determining a bat's trophic level requires extensive field observations and analysis. Due to these complexities, trophic levels have been documented for only 423 species: 101 herbivores, 259 carnivores, and 63 omnivores. While we would not use this trophic information for clustering bat species, we explore if the impact of precipitation on forearm length is a key factor that influences the evolutionary dietary patterns of bats, or, if the dietary pattern of bats might evolve in a mutually influential way with how they adapt to the impact of precipitation on the development of their forearm lengths.

#### 5.1.1. Model fitting and hypothesis tests results

We regard forearm length as the response variable and precipitation as covariate and fit the Chiroptera data to GM, Homo-GMR, and GMR models. Without prior knowledge of the order of the mixture, we fit these models with orders between 2 to 8. Table 11 presents the p-values associated with the proposed tests concerning the impact of precipitation on forearm length. The small p-values from the overall test suggest a significant precipitation effect on bat's forearm length when there are mixture of subpopulations of bat species in the sample. The small p-values of heterogeneity effects tests suggest a strong evidence that the precipitation has different effects on the forearm length development depending on which subpopulation the bat is from. These results suggest that bat's forearm development evolved differently in response to the precipitation.

**Table 11.** P-values associated with the overall and heterogeneity effects tests for precipitation effects, and the optimal model selected in sequential test, based on the four testing procedures. Sequential test has the same significance levels as the overall test and the heterogeneity effects test.

Procedure	Overall test	Heterogeneity effects test	Best model by sequential test
Naive I	$2.233*10^{-13}$	$3.575*10^{-12}$	5-GMR
Naive II	$6.255*10^{-8}$	$4.409*10^{-7}$	7-GMR
Naive III	$2.233*10^{-13}$	$3.575*10^{-12}$	5-GMR
WEST	$2.540*10^{-7}$	$3.494*10^{-7}$	5-GMR

Consequently, we determine the order of the GMR model from each of the 4 testing procedures. Following the Naive I, III, and WEST procedures, an order of 5-component GMR model is preferred, while following the Naive II procedure, an order of 7-component GMR model is preferred. Naive II procedure tends to select the order for the model favoring the null hypothesis in general. However, as the null hypotheses of both the overall and heterogeneity test are rejected by all four testing procedures, the model selected by Naive II procedure might introduce bias.

**Figure 2.**
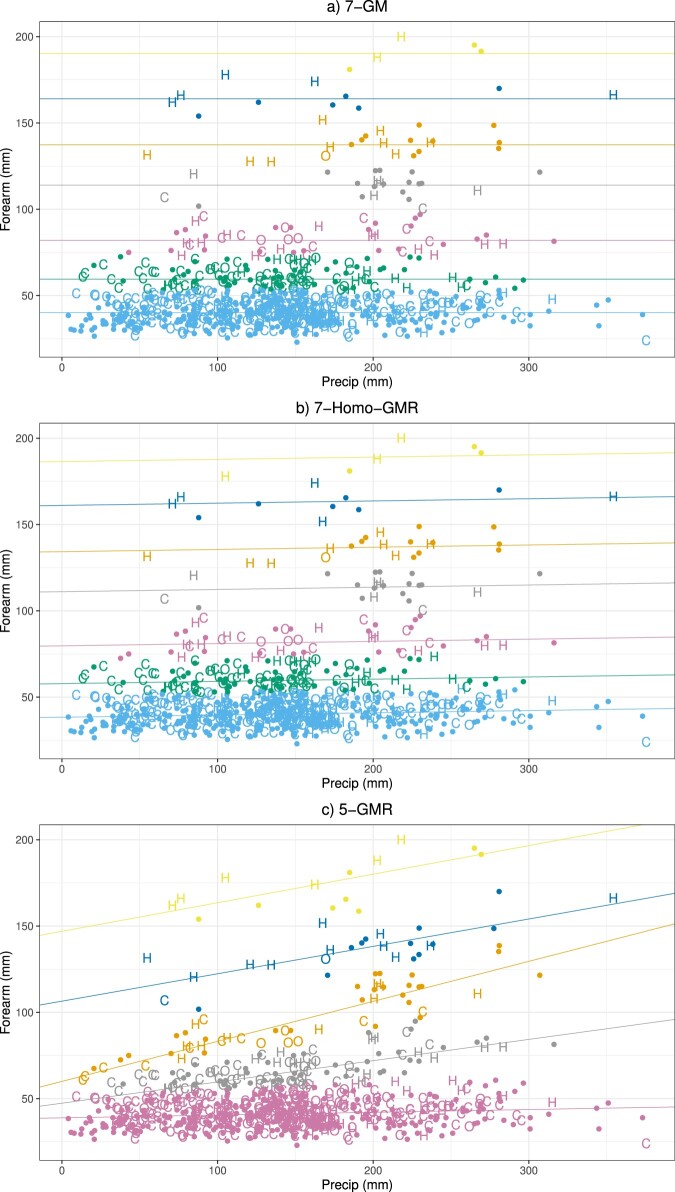
Scatterplots of Chiroptera data clustered by 7-GM, 7-Homo-GMR and 5-GMR models, respectively, where colors refer to the fitted groups, letters in plot refer to the classified groups: H (herbivore,), C (carnivore), and O (omnivore), and unmarked dots refer to the species that lack information on trophic level.

Table 12 lists the BIC values for all fitted models. In Figure 2, we present the scatterplots of precipitation against forearm length using the model with the minimum BIC from each of the three types of mixture models. Figure 2(a) displays the clustering analysis result from the 7-component GM model, where the clustering of bats uses only the response variable forearm length without using the precipitation variable. Figure 2(b) presents the results from the 7-component Homo-GMR model. It assumes a homogeneous effect of precipitation on the forearm length across all clusters. Figure 2(c) shows the clustering result of 5-component GMR model, where the influence of precipitation varies among clusters. The distinct slope of the regression line for each cluster signifies these differences: the pink cluster at the bottom of the plot indicates a very low effect of the precipitation on the forearm length; other clusters demonstrate that as precipitation increases, forearm length also tends to increase, though the level of this effect varies among the clusters. The GM and Homo-GMR models cluster the samples similarly. This result is further corroborated by the parameter estimates, π,β and *σ*, for each optimal model, as detailed in Table 13 that the estimated values are quite similar between the GM and Homo-GMR model, and the fitted regression coefficient ${\text{β}}_{1g}$ of the Homo-GMR model is very closed to zero. The two models also lead to similar membership assignments for each subject. With the 5-component GMR model, 74% bats are assigned to the cluster at the bottom of the plot in Figure 2(c), where bats generally with shorter forearm length and the precipitation has a very mild effect on the forearm length. Notably, for the third cluster from the top, the precipitation has the strongest effect on forearm length ( ${\text{β}}_{13}=0.23$).

**Table 12.** BICs values of fitted models for the Chiroptera data.

Model	*G* = 2	*G* = 3	*G* = 4	*G* = 5	*G* = 6	*G* = 7	*G* = 8
GM	7381.18	7280.00	7172.45	7162.15	7143.03	7132.84	7137.87
Homo-GMR	7382.06	7282.41	7171.58	7160.08	7143.04	7132.89	7135.56
GMR	7388.66	7259.22	7172.10	7127.44	7137.89	7133.22	7130.07

**Table 13.** Estimated parameters of 7-component GM, 7-component Homo-GMR, and 5-component GMR models for the Chiroptera data.

Parameter	7-GM
$\pi_{g}$	(0.01, 0.01, 0.02, 0.03, 0.06, 0.18, 0.69)
$\mu_{g}$	(190.12, 164.00, 137.34, 114.02, 82.01, 59.54, 40.15)
*σ*	7.06
Parameter	7-Homo-GMR
$\pi_{g}$	(0.01, 0.01, 0.02, 0.03, 0.06, 0.18, 0.69)
${\text{β}}_{0g}$	(186.46, 161.08, 134.27, 111.09, 79.73, 57.84, 38.37)
${\text{β}}_{1g}$	(0.01, 0.01, 0.01, 0.01, 0.01, 0.01, 0.01)
*σ*	7.01
Parameter	5-GMR
$\pi_{g}$	(0.02, 0.03, 0.06, 0.15, 0.74)
${\text{β}}_{0g}$	(146.96, 106.50, 60.03, 47.39, 38.91)
${\text{β}}_{1g}$	(0.17, 0.16, 0.23, 0.12, 0.02)
*σ*	8.03

We further explore whether the impact of precipitation on forearm length might influence the bats' dietary patterns over evolutionary time. In Figure 2(c), we label the species accordingly if their trophic levels are known, otherwise, they appear as dots in the plot. Under the 5-component GMR model, species in the first two top clusters are in yellow and blue colors. If their tropic levels are known, they are mostly herbivores. There is a positive association between precipitation levels and the forearm length in those two subpopulations. We can possibly explain this result as in regions with higher precipitation, there might be higher availability and diversity of fruits and flowers. The herbivorous bats would require longer forearms to efficiently fly over a larger area to forage and carry food to their roosts. Most carnivores and omnivores are in the lower clusters, indicated by grey and pink colors in which the precipitation has a lower effect on the forearm length. Carnivorous and omnivorous bats generally prey on insects or others that have a more varied diet such that their prey availability might not be as directly influenced by precipitation as plants for herbivores. This potentially reduces the natural drive for evolutionary adaptations in forearm length in response to changes in precipitation.

### 5.2. Sensitivity analysis

The results from model-based clustering methods are sensitive to various factors such as outliers, the inclusion or exclusion of some arbitrary sample points, and the sample size. Therefore, we perform the sensitivity analysis on the Chiroptera to evaluate the robustness of our four proposed testing procedures on the three hypothesis tests.

For the Chiroptera data, sample points were removed in three ways: 1)removing 416 out of 839 species that missed the trophic information to have a completed trophic information dataset (n= 423); 2) randomly removing 5% of the species (*n* = 797); and 3) randomly removing 10% of the species (*n* = 755). Table 14 shows that the p-values of overall and heterogeneous effect tests using the three reduced datasets of Chiroptera data are all very low, which indicates the precipitation has a significant effect on bat's forearm length and this effect is heterogeneous across all subpopulations. The p-values are higher when using the tropic completed dataset. It is probably due to a much reduced sample size. During the sequential test, Naive I, III and WEST procedures suggest the 5-component GMR model as the best fitting model for all subsets of data. We observe consistency in both the clustering and hypothesis testing results when using the original dataset and the reduced datasets. See Figure S1 in the Supplementary Materials for clustering result comparisons. The Naive II procedure suggests the 4-component GMR model for the tropic completed dataset and the 7-component GMR model for the other two randomly reduced datasets. As discussed in Section 5.1.1, Naive II procedure is likely to prefer the incorrect model, and is more sensitive to the change of the data as well.

**Table 14.** The p-values of overall test and heterogeneous effect test using naive procedures and weighted significance test procedure, and the optimal model selected using sequential test, on trophic-complete data, 5% off data and 10% off data.

Overall test			
Data (sample size)	Naive I, III	Naive II	WEST
Trophic-complete (n=423)	$6.389*10^{-7}$	$1.247*10^{-3}$	$2.064*10^{-3}$
95% of data (n=797)	$1.834*10^{-12}$	$2.820*10^{-7}$	$4.735*10^{-7}$
90% of data (n=755)	$2.928*10^{-10}$	$1.471*10^{-6}$	$2.281*10^{-6}$
Heterogeneous effect test			
Data (sample size)	Naive I, III	Naive II	WEST
Trophic-complete (n=423)	$3.045*10^{-7}$	$4.557*10^{-4}$	$9.510*10^{-4}$
95% of data (n=797)	$1.875*10^{-11}$	$1.938*10^{-6}$	$1.745*10^{-6}$
90% of data (n=755)	$9.596*10^{-10}$	$3.122*10^{-6}$	$3.739*10^{-6}$
Sequential test Best model			
Data (sample size)	Naive I, III	Naive II	WEST
Trophic-complete (n=423)	5-GMR	4-GMR	5-GMR
95% of data (n=797)	5-GMR	7-GMR	5-GMR
90% of data (n=755)	5-GMR	7-GMR	5-GMR

## 6. Summary and discussions

Mixture regression models have been applied to real world problems in various areas. In model-based clustering, mixture regression models are used to cluster observations into subgroups and capture heterogeneity among subgroups by the tailored regression model for each subgroup. It is common that a set of covariates of interest are considered and modeled using mixture regression models where their effects are allowed to be varied across components. However, it is possible that not all covariates in the model are relevant to the response, and thus some should be omitted from the model. Khalili and Chen in [9] proposed a variable selection method in finite mixture regression models by introducing a new class of penalties which can filter out variables of no importance. Our proposed method would provide a p-value for the significance of a covariate, which can be further developed as a variable selection tool that is based on hypothesis tests, e.g. forward selection when there is more than one covariate.

When testing the association between a covariate and the response, the likelihood ratio test is often used. The LRT statistic asymptotically follows a chi-square distribution with the degrees of freedom linked to the order of the mixture regression model. In practice, the order is unknown. This poses challenges in the inference procedure for testing the association between the covariates and the response [3]. Chen et al. [4] proposed and developed a likelihood-based EM test for the order of GM models. It is worth investigating the possibility of including such an inference step in our proposed testing procedures.

When a covariate is known to have an effect on the response, or following the decision of rejecting the null hypothesis that a covariate has no effect on the response, a natural question of whether the covariate has the same effect on the response across all subgroups or not will be asked. In this paper, we consider two different hypotheses regarding the association between a covariate and the response within the mixture models: 1) the overall effect, and 2) the heterogeneous effect. Testing the heterogeneity of a covariate's effect on the response is important when the sample is a constitution of multiple groups. Woolf's test [18] was the first in application to test the homogeneity of the odds ratios among multiple groups. Yilmaz [19] studied the reliabilities and the accuracies for testing the homogeneity of odd ratios among multiple groups. Armistead [1] discussed the pitfalls in assessing association of categorical variables and particularly, the diagnostic likelihood ratio measure with imbalanced data. While testing the homogeneity of odds (or log-odds) ratio is equivalent to testing the homogeneous effect of a categorical covariate on a binary outcome among multiple groups, our proposed heterogeneous effect test (b) is somewhat similar to those but for continuous response under GMR model rather than binary outcomes. In addition, the order of the mixture (or the number of groups) and memberships of observations are unknown and required to be estimated. In this paper, our proposed testing procedures are all likelihood ratio based. It is worth developing non-likelihood ratio based procedures for the proposed hypothesis tests under mixture regression models. We propose Nave I, Nave II, Nave III, and WEST procedures for these hypotheses adaptive to order selections. The Nave I, II and III procedures are somewhat ad hoc while WEST procedure based on the Bayesian approach is more rigorous in the sense as it focuses on testing the association between the covariate and the response without a data adaptive order. Comparing the type I error rate, power of the test, and the accuracy of the clustering analysis, WEST procedure has a better performance overall. In the analysis of the Chiroptera dataset, GMR model is used to capture various types of linear relationships within the underlying bat subpopulations. The proposed test better addresses the Chiroptera data, with p-values provided to evaluate the significance of precipitation in both the overall and the heterogeneous effect tests. Our results reveal that precipitation has different effects on the forearm length development across these subpopulations over evolutionary time. For demonstration and application, we also applied our proposed methods to the diabetes data, in which, the association between response, three hour oral glucose tolerance, and covariate, steady state plasma glucose, under the GMR model will be tested. We perform the overall effect test, heterogeneous effect test, and sequential test. Please see Section 4.2 in the Supplementary Materiel for the results.

In this paper, we consider one covariate. However, the proposed procedures are applicable to mixture regression concerning effect patterns (homogeneous vs heterogeneous) of multiple covariates. These patterns, however, lead to complex constraints on regression coefficients across subpopulations. We find it challenging to work on the corresponding constrained MLEs. Furthermore, our idea can be applied to other distributional mixture regression models. For example, distributions in the exponential family can be used to accommodate response variables of other data types such as count data.

In our simulation study, for assessing the type I error rate of the overall test, datasets were simulated under the null hypothesis that *X* has no effect on *Y* in all subpopulations. The empirical rejection rates slightly increase with decreasing sample size, while remaining close to the nominal significance level. The empirical type I error rate and the percentage of correctly selected models complement each other with their sum close to 1 for all scenarios and using all proposed methods. We also consider practical situations when the response variable is influenced by an unobserved covariate where an irrelevant covariate is being tested. From the results of the simulation study, we found that the Nave I and III methods are less stable in controlling the type I error rates. In terms of power assessment, the performances are quite similar among all four procedures across all scenarios. When assessing the percentage of correctly selected models, there are two sources of variation that contribute to the performance of the proposed testing procedures: the rejection decision of the hypothesis test and the order specification of the mixture model. For example, Table S2 in the Supplementary Materials summarizes the simulation study results for the sequential test when the data is generated under $H_{0}$ of a 3-component Homo-GMR model. In Scenario 1 where the three clusters are well separated, the values of the percentages of correctly selected models are close to the power of overall test multiplied by the (1 – Type I error rate) of the heterogeneous effect test. When the three clusters are not well separated as in Scenario 2 with sample size of 300, the values of the percentages of correctly selected models are much less than the value of the power of the overall test multiplied by the (1 – Type I error rate) of the heterogeneous effect test, as the procedures tend to select 2-component Homo-GMR rather than the 3-component Homo-GMR. One can argue that the percentage of the correctly selected model is the ultimate evaluation criterion for the performance of an inference procedure for the precision of clustering. However, it is also important to develop a practical procedure to help make inference decisions on the relationship between a covariate and the response under the mixture regression model framework. Overall, the weighted significance test procedure generally outperforms the others, especially when sample size is large, although it might not be the winner for all scenarios and in all evaluation metrics.

## Supplementary Material

GMR_Suppl.pdf

## Supplementary Materials

S1. EM algorithm for finding restricted MLE of β under Homo-GMR model
S2. Weighted significance test
S3. Simulation Studies
  S3.1 Models and results of simulation study under equal variance assumptionTable S1-S9
  S3.2 Models and results of simulation study without equal variance assumptionTable S10-S18
S4. Additional information in real data analysis
  S4.1 Application to Chiroptera dataFigure S1
  S4.2 Application to diabetes dataTable S19-20Figure S2
S5. Source Code
