# Supplementary material for: Tests of covariate effects under finite Gaussian mixture regression models
Source: J Appl Stat. 2024 Nov 27;52(8):1571–93. doi: 10.1080/02664763.2024.2433567 (PMC12147513; doi:10.1080/02664763.2024.2433567)
Supplement: GMR_Suppl.pdf [file CJAS_A_2433567_SM0554.pdf]

Supplementary Materials for "Tests of Covariate Effects under Finite Gaussian Mixture Regression Models"

Chong Gan[a], Jiahua Chen[b] and Zeny Feng[a]

[a]Department of Mathematics and Statistics, University of Guelph, Guelph, Canada;
[b]Department of statistics, University of British Columbia, Vancouver,Canada.

ARTICLE HISTORY
Compiled November 5, 2024

1. EM algorithm for finding restricted MLE of $\boldsymbol{\beta}$ under Homo-GMR model

In Homo-GMR model with one covariate, the regression coefficients β_{1g} of X are equal across all subpopulations, i.e. $\beta_{11} = \beta_{12} = \cdots = \beta_{1G} = \beta_1$. Under model assumption $\sigma_g^2 = \sigma^2$ for all g, the incomplete data log likelihood function for the G-component Homo-GMR model is given by

$$\begin{aligned}\ell(\pi_g, \boldsymbol{\beta}_g, \sigma^2) &= \sum_{i=1}^{n} \log\{\sum_{g=1}^{G} \pi_g \phi(y_i; x_i, \beta_{0g}, \beta_1, \sigma^2)\} \\ &= \sum_{i=1}^{n} \log\{\sum_{g=1}^{G} \pi_g (2\pi\sigma^2)^{-\frac{1}{2}} \exp\left\{-\frac{(y_i - \beta_{0g} - \beta_1 x_i)^2}{2\sigma^2}\right\}\}.\end{aligned}$$

and the complete data log likelihood function is given as

$$\begin{aligned}\ell_c(\pi_g, \boldsymbol{\beta}_g, \sigma^2) &= \sum_{i=1}^{n}\sum_{g=1}^{G} Z_{ig} \log\{\pi_g \phi(y_i; x_i, \beta_{0g}, \beta_1, \sigma^2)\} \\ &= \sum_{i=1}^{n}\sum_{g=1}^{G} Z_{ig} \log\{\pi_g (2\pi\sigma^2)^{-\frac{1}{2}} \exp\left\{-\frac{(y_i - \beta_{0g} - \beta_1 x_i)^2}{2\sigma^2}\right\}\}\end{aligned}$$

Given the initial values of $\pi^{(0)}, \boldsymbol{\beta}^{(0)}$, and $\sigma^{2(0)}$, in the E-step of the 1st iteration, the conditional expectation of the complete data log-likelihood is found to be

CONTACT Zeny Feng. Email: zfeng@uoguelph.ca

$$
\begin{aligned}
Q(\pi, \boldsymbol{\beta}, \sigma^2 | \pi^{(0)}, \boldsymbol{\beta}^{(0)}, \sigma^{2(0)}) &= E\left\{ \sum_{i=1}^{n} \sum_{g=1}^{G} Z_{ig} \log[\pi_g \phi(y_i; x_i, \beta_{0g}, \beta_1, \sigma^2)] | \pi^{(0)}, \boldsymbol{\beta}^{(0)}, \sigma^{2(0)} \right\} \\
&= \sum_{i=1}^{n} \sum_{g=1}^{G} E(Z_{ig} | \pi^{(0)}, \boldsymbol{\beta}^{(0)}, \sigma^{2(0)}, y_i, x_i) \log\{\pi_g \phi(y_i; x_i, \beta_{0g}, \beta_1, \sigma^2)\} \\
&= \sum_{i=1}^{n} \sum_{g=1}^{G} Z_{ig}^{(1)} \log\{\pi_g \phi(y_i; x_i, \beta_{0g}, \beta_1, \sigma^2)\}
\end{aligned}
$$

and the conditional expectation $Z_{ig}^{(1)}$ are given by

$$
Z_{ig}^{(1)} = \frac{\pi_g^{(0)} \phi(y_i; x_i, \beta_{0g}^{(0)}, \beta_1^{(0)}, \sigma^{2(0)})}{\sum_{g=1}^{G} \pi_g^{(0)} \phi(y_i; x_i, \beta_{0g}^{(0)}, \beta_1^{(0)}, \sigma^{2(0)})}
$$

In the M-step, we maximize Q function with respect to π_g, β_1, β_{0g} which leads to

$$
\begin{aligned}
\pi_g^{(1)} &= n^{-1} \textstyle\sum_{g=1}^{G} Z_{ig}^{(1)}, \\
\beta_1^{(1)} &= \frac{\sum_{i=1}^{n} x_i \sum_{g=1}^{G} Z_{ig}^{(1)} (y_i - \bar{y}_g^{(1)})}{\sum_{i=1}^{n} x_i \sum_{g=1}^{G} Z_{ig}^{(1)} (x_i - \bar{x}_g^{(1)})}, \\
\beta_{0g}^{(1)} &= \bar{y}_g^{(1)} - \beta_1^{(1)} \bar{x}_g^{(1)}, \\
\sigma^{2(1)} &= n^{-1} \textstyle\sum_{i=1}^{n} \sum_{g=1}^{G} Z_{ig}^{(1)} (y_i - \beta_{0g}^{(1)} - \beta_1^{(1)} x_i)^2
\end{aligned}
$$

where $\bar{y}_g^{(1)} = \frac{\sum_{i=1}^{n} Z_{ig}^{(1)} y_i}{\sum_{i=1}^{n} Z_{ig}^{(1)}}$ and $\bar{x}_g^{(1)} = \frac{\sum_{i=1}^{n} Z_{ig}^{(1)} x_i}{\sum_{i=1}^{n} Z_{ig}^{(1)}}$ for $g = 1, ..., G$. Note that, the solution for β_{0g}, β_1, and σ^2 are analogue to the weighted least square estimates of those in a simple linear regression model.

2. Weighted significance test

Let the set of competing models be $M = \{M_1, M_2, ..., M_J\}$. Each M_j is a particular family of distributions denoted as

$$
M_j = \{f_j(y; \boldsymbol{\theta}_j); \boldsymbol{\theta}_j \in \boldsymbol{\Theta}_j \subset \mathcal{R}^{p_j}\}
$$

with the dimension of the parameter p_j. In the Bayesian context, one places a prior distribution in the form of $\sum_{j=1}^{J} \alpha_j \mathbb{p}_j$ so that α_j is the prior probability of model M_j and $\mathbb{p}_j$ is a prior distribution on Θ_j.

Let Y be a sample of size n from a population with the distribution function being a member of model in M. Then, the posterior probability of M_j conditional on the observed data y is proportional to post(M_j) as specified in [**?**] and post(M_j) is given by

$$
\text{post}(M_j) = \alpha_j \int f_j(y; \boldsymbol{\theta}_j) \mathbb{p}_j(\boldsymbol{\theta}_j) d\boldsymbol{\theta}_j.
$$

When the sample size n is large so that the Laplace approximation is sufficiently

precise, the post(M_j) is well approximated by

$$\text{post}(M_j) \approx (2\pi)^{-p_j/2}\alpha_j f_j(y; \hat{\boldsymbol{\theta}}_j)|\mathcal{I}_n(\hat{\boldsymbol{\theta}}_j)|^{-1/2},$$

where $\hat{\boldsymbol{\theta}}_j$ is the MLE of $\boldsymbol{\theta}_j$, $\mathcal{I}_n(\cdot)$ is observed the Fisher information matrix for $\boldsymbol{\theta}_j$ under the model M_j. With an independent identically distributed random sample conditional on the given covariate value, $|\mathcal{I}_n| = c_j n^{p_j}$, where c_j's are some constant depending on the f_j's. Hence, by ignoring terms whose sizes are $O_p(1)$, we get

$$-2\log\{\text{post}(M_j)\} \approx -2\ell(\hat{\boldsymbol{\theta}}_j; M_j) + p_j \log n$$

where $\ell(\cdot; M_j) = \log f_j(y; \cdot)$ is the log likelihood under model M_j. Based on this approximation, it is suggested to define

$$\text{BIC}(M_j) = -2\ell(\hat{\boldsymbol{\theta}}_j; M_j) + p_j \log n.$$

If one is asked to select a model to fit the data according to the Bayes rule, a model M_j is selected if M_j has the highest posterior, i.e, post(M_j) is the maximal among all post$(M_{j'})$ for all $j' = 1, ..., J$. Let

$$A_j = (2\pi)^{-p_j/2}\alpha_j f_j(y_n; \hat{\boldsymbol{\theta}}_j)|\mathcal{I}_n(\hat{\boldsymbol{\theta}}_j)|^{-1/2}$$

we may approximate the posterior probability of model M_j among J candidate models by

$$\Pi_j \approx \frac{A_j}{\sum_{j=1}^{J} A_j},$$

which is approximately

$$\Pi_j \approx \frac{\exp(-\frac{1}{2}BIC(M_j))}{\sum_{j=1}^{J} \exp(-\frac{1}{2}BIC(M_j))}$$

Suppose a hypothesis test is formulated such that it is applicable to all competing models. For example, under each model M_j, we may test the existence of an overall effect of a covariate X. Let T_j be the test statistics under M_j, and t_j be its observed value. A p-value specific to M_j can be defined as

$$\text{p-value} = P(T_j \geq t_j | H_0, M_j).$$

When an LRT is employed, we have asymptotically T_j =LRT$(M_j) \sim \chi^2_{k_j}$ under H_0 with k_j being the difference of the numbers of parameters under H_a and H_0. By the rule of total probability, we obtain a weighted significance level in the form

$$p_0 = \sum_{j=1}^{J} P(T_j \geq t_j | H_0, M_j)\Pi_j.$$

Hence, p_0 is a suitable metric to measure statistical significance.

3. Additional simulation results

3.1. Models and results of simulation study under equal variance assumption

Table S1. True values of parameters in simulation study for sequential test

**Homogeneous effect**					
**Scenario**	π	$\boldsymbol{\beta}_1$	$\boldsymbol{\beta}_2$	$\boldsymbol{\beta}_3$	σ
1	(0.3, 0.5, 0.2)	(5, 0.3)	(1, 0.3)	(-3, 0.3)	1
2	(0.3, 0.5, 0.2)	(3, 0.3)	(-1, 0.3)	(-3, 0.3)	1
3	(0.3, 0.5, 0.2)	(5, 0.3)	(1, 0.3)	(-3, 0.3)	$\sqrt{2}$
**Heterogeneous effect**					
**Scenario**	π	$\boldsymbol{\beta}_1$	$\boldsymbol{\beta}_2$	$\boldsymbol{\beta}_3$	σ
1	(0.3, 0.5, 0.2)	(5, 0)	(1, 0.2)	(-3, 0.5)	1
2	(0.3, 0.5, 0.2)	(3, 0)	(-1, 0.2)	(-3, 0.5)	1
3	(0.3, 0.5, 0.2)	(5, -0.2)	(1, 0)	(-3, 0.1)	$\sqrt{2}$

Table S2. The power (overall test) and type I error rate (heterogeneous effect test) of sequential test at $\alpha = 0.05$, percentage of correctly selected models, and mean of adjusted rand indices. Datasets were simulated under the scenarios that x has homogeneous effect on the responses y (Table S1) when $n = 500$. The Naive I, II, III and WEST procedures were performed.

**Scenario**		**Naive I**	**Naive II**	**Naive III**	**WEST**
1	Power (Overall test)	0.991	0.991	0.990	0.991
	Type I error rate (Heterogeneous test)	0.054	0.054	0.055	0.054
	% of correct models	0.935	0.936	0.934	0.934
	Mean ARI	0.881	0.881	0.881	0.881
2	Power (Overall test)	0.977	0.977	0.973	0.976
	Type I error rate (Heterogeneous test)	0.057	0.052	0.058	0.045
	% of correct models	0.604	0.649	0.571	0.786
	Mean ARI	0.625	0.631	0.624	0.646
3	Power (Overall test)	0.661	0.660	0.594	0.641
	Type I error rate (Heterogeneous test)	0.092	0.086	0.099	0.081
	% of correct models	0.567	0.571	0.489	0.575
	Mean ARI	0.639	0.640	0.623	0.650

Table S3. The power (overall test and heterogeneous effect test) of sequential test at $\alpha = 0.05$, percentage of correctly selected models, and mean of adjusted rand indices. The datasets were simulated under the scenarios that x has heterogeneous effect on the responses y (Table S1) when $n = 500$. The Naive I, II, III and WEST procedures were performed.

Scenario		Naive I	Naive II	Naive III	WEST
1	Power (Overall test)	0.977	0.977	0.970	0.978
	Power (Heterogeneous test)	0.882	0.882	0.887	0.883
	% of correct models	0.862	0.858	0.860	0.864
	Mean ARI	0.895	0.895	0.895	0.895
2	Power (Overall test)	0.933	0.933	0.923	0.934
	Power (Heterogeneous test)	0.816	0.815	0.817	0.799
	% of correct models	0.660	0.711	0.630	0.623
	Mean ARI	0.668	0.677	0.663	0.666
3	Power (Overall test)	0.244	0.244	0.200	0.232
	Power (Heterogeneous test)	0.783	0.783	0.795	0.763
	% of correct models	0.185	0.186	0.148	0.166
	Mean ARI	0.658	0.661	0.649	0.653

Table S4. Type I error rate of overall test at $\alpha = 0.01$, percentage of correctly selected models, and mean of adjusted rand indices. Datasets were simulated under H_0 that X has no effect on the responses Y under equal variance assumption when $n = 500$. The Naive I, II, III and weighted significance test procedures were performed.

Scenario		Naive I	Naive II	Naive III	WEST
1	Type I error rate	0.203	0.023	0.147	0.013
	% of correct models	0.147	0.169	0.196	0.213
	Mean ARI	0.175	0.072	0.174	0.104
2	Type I error rate	0.020	0.020	0.016	0.016
	% of correct models	0.844	0.844	0.845	0.848
	Mean ARI	0.996	0.996	0.996	0.996
3	Type I error rate	0.010	0.010	0.010	0.010
	% of correct models	0.008	0.008	0.008	0.008
	Mean ARI	0.643	0.643	0.643	0.643

Table S5. Power of overall test at $\alpha = 0.01$, percentage of correctly selected models, and mean of adjusted rand indices. Datasets were simulated under H_a that X has effect on the responses Y under equal variance assumption when $n = 500$. The naive I, II, III and weighted significance test procedures were performed.

Scenario		Naive I	Naive II	Naive III	WEST
1	Power	1	1	1	1
	% of correct models	0.877	0.338	0.877	0.877
	Mean ARI	0.772	0.726	0.772	0.772
2	Power	0.998	0.997	0.998	0.998
	% of correct models	0.997	0.861	0.997	0.997
	Mean ARI	1.000	0.999	1.000	1.000
3	Power	0.437	0.437	0.433	0.434
	% of correct models	0.003	0.005	0.001	0.001
	Mean ARI	0.648	0.648	0.648	0.648

Table S6. Type I error rate of heterogeneous effect test at $\alpha = 0.01$, percentage of correctly selected models, and mean of adjusted rand indices. Datasets were simulated under H_0 that X has homogeneous effect on the responses Y under equal variance assumption when $n = 500$. The Naive I, II, III and weighted significance test procedures were performed.

Scenario		Naive I	Naive II	Naive III	WEST
1	Type I error rate	0.007	0.007	0.007	0.007
	% of correct models	0.990	0.990	0.990	0.990
	Mean ARI	0.916	0.916	0.916	0.916
2	Type I error rate	0.013	0.012	0.013	0.012
	% of correct models	0.981	0.982	0.981	0.982
	Mean ARI	0.768	0.768	0.768	0.768
3	Type I error rate	0.011	0.011	0.011	0.010
	% of correct models	0.085	0.085	0.085	0.085
	Mean ARI	0.459	0.459	0.459	0.459

Table S7. Power of heterogeneous effect test at $\alpha = 0.01$, percentage of correctly selected models, and mean of adjusted rand indices. Datasets were simulated under H_a that X has heterogeneous effect on the responses Y under equal variance assumption when $n = 500$. The Naive I, II, III and weighted significance test procedures were performed.

Scenario		Naive I	Naive II	Naive III	WEST
1	Power	0.960	0.960	0.960	0.960
	% of correct models	0.959	0.947	0.959	0.957
	Mean ARI	0.913	0.913	0.913	0.913
2	Power	0.086	0.086	0.086	0.082
	% of correct models	0.085	0.086	0.085	0.083
	Mean ARI	0.766	0.766	0.766	0.766
3	Power	0.508	0.503	0.507	0.489
	% of correct models	0.042	0.054	0.040	0.036
	Mean ARI	0.449	0.451	0.449	0.449

Table S8. The power (overall test) and type I error rate (heterogeneous effect test) of sequential test at $\alpha = 0.01$, percentage of correctly selected models, and mean of adjusted rand indices. Datasets were simulated under the scenarios that X has homogeneous effect on the responses Y under equal variance assumption when $n = 500$. The Naive I, II, III and weighted significance test procedures were performed.

**Scenario**		**Naive I**	**Naive II**	**Naive III**	**WEST**
1	Power (Overall test)	0.971	0.971	0.971	0.971
	Type I error rate (Heterogeneous test)	0.013	0.013	0.013	0.013
	% of correct models	0.956	0.956	0.956	0.955
	Mean ARI	0.880	0.880	0.880	0.880
2	Power (Overall test)	0.932	0.931	0.925	0.927
	Type I error rate (Heterogeneous test)	0.012	0.012	0.012	0.012
	% of correct models	0.600	0.644	0.579	0.772
	Mean ARI	0.624	0.631	0.620	0.646
3	Power (Overall test)	0.421	0.420	0.397	0.399
	Type I error rate (Heterogeneous test)	0.043	0.033	0.043	0.033
	% of correct models	0.379	0.383	0.352	0.381
	Mean ARI	0.639	0.639	0.631	0.654

Table S9. The power (overall test and heterogeneous effect test) of sequential test at $\alpha = 0.01$, percentage of correctly selected models, and mean of adjusted rand indices. The datasets were simulated under the scenarios that X has heterogeneous effect on the responses Y under equal variance assumption when $n = 500$. The Naive I, II, III and weighted significance test procedures were performed.

**Scenario**		**Naive I**	**Naive II**	**Naive III**	**WEST**
1	Power (Overall test)	0.922	0.922	0.918	0.920
	Power (Heterogeneous test)	0.745	0.744	0.748	0.745
	% of correct models	0.687	0.683	0.687	0.685
	Mean ARI	0.895	0.894	0.895	0.895
2	Power (Overall test)	0.829	0.829	0.819	0.824
	Power (Heterogeneous test)	0.666	0.663	0.664	0.606
	% of correct models	0.486	0.524	0.473	0.430
	Mean ARI	0.668	0.677	0.663	0.666
3	Power (Overall test)	0.087	0.087	0.084	0.085
	Power (Heterogeneous test)	0.747	0.747	0.750	0.729
	% of correct models	0.064	0.065	0.061	0.060
	Mean ARI	0.669	0.677	0.661	0.667

3.2. Models and results of simulation study without equal variance assumption

When the variances across subpopulations are allowed to be different, the log likelihood function of $\boldsymbol{\Theta}$ under GMR model can be reformulated as

$$\ell(\boldsymbol{\Theta}) = \sum_{i=1}^{n} \log\{\sum_{g=1}^{G} \pi_g (2\pi\sigma_g^2)^{-\frac{1}{2}} \exp\left\{ -\frac{(y_i - \boldsymbol{\beta}_g^\top \boldsymbol{x}_i)^2}{2\sigma_g^2} \right\}\}.$$

The log likelihood function $\ell(\boldsymbol{\Theta})$ is unbounded, i.e. $\sup_{\boldsymbol{\Theta}} \ell(\boldsymbol{\Theta}) = \infty$. The infinite supremum is attained, for instance, when $y_i = \boldsymbol{\beta}_g^\top \boldsymbol{x}_i$ and $\sigma_g \to 0$. More discussions can be found in [?] and [?]. In practice, σ_g or $\hat{\sigma}_g = 0$ would imply that there is a subpopulation consisting of only one observation in the population or in the sample, which is very unrealistic. "mixtools" [?] is a R package commonly used for analyzing finite mixture models. It reports that the algorithm runs into such special scenarios infrequently. When such cases arise, "mixtools" functions rerun the EM algorithms using different initial values, which is also typical as EM algorithm often yields a local maximum. R package "FlexMix" [?] also obtains MLEs of parameters based on the same log likelihood while allowing for multiple initial values and removing subpopulations with extremely low weights. R package "MixtureInf" [?] employs a penalty to avoid this problem following [?]. In order to investigate the robustness of our testing procedures with equal variance assumption, we considered additional simulation settings with unequal σ_g values. See Table S10 to S13 for parameter settings in several scenarios. We use EM algorithm to obtain values of $\hat{\boldsymbol{\Theta}}_{\text{GM}}$, $\hat{\boldsymbol{\Theta}}_{\text{Homo-GMR}}$ and $\hat{\boldsymbol{\Theta}}_{\text{GMR}}$ without equal variance assumption and perform hypothesis tests, with the strategy of re-start the algorithm with different initial values if a fit has degenerate subpopulation variances.

Table S10. True values of parameters in simulation study to assess the type I error rate for overall test, without equal variance assumption.

Overall test under H_0					
**Scenario**	π	$\boldsymbol{\beta}_1$	$\boldsymbol{\beta}_2$	$\boldsymbol{\beta}_3$	σ
1	(0.15, 0.1, 0.75)	(1549, 0)	(987, 0)	(332, 0)	(60, 70, 80)
2	(0.15, 0.1, 0.75)	(1549, 0)	(1250, 0)	(332, 0)	(60, 70, 80)
3	(0.15, 0.1, 0.75)	(1549, 0)	(987, 0)	(332, 0)	(200, 250, 300)

Table S11. True values of parameters in simulation study to assess the power for overall test, without equal variance assumption.

Parameter settings for the overall test					
**Scenario**	π	$\boldsymbol{\beta}_1$	$\boldsymbol{\beta}_2$	$\boldsymbol{\beta}_3$	σ
1	(0.15, 0.1, 0.75)	(1549, -3.82)	(987, -1.31)	(332, 0.30)	(60, 70, 80)
2	(0.15, 0.1, 0.75)	(1549, -0.382)	(987, -0.131)	(332, 0.03)	(60, 70, 80)
3	(0.15, 0.1, 0.75)	(1549, -0.764)	(987, -0.262)	(332, 0.06)	(250, 350, 300)

Table S12. True values of parameters in simulation study for heterogeneous effect test, without equal variance assumption.

**Parameter settings under H_0 of homogeneous covariate effects**					
**Scenario**	π	$\boldsymbol{\beta}_1$	$\boldsymbol{\beta}_2$	$\boldsymbol{\beta}_3$	σ
1	(0.5, 0.3, 0.2)	(5, 1)	(1, 1)	(-3, 1)	(0.7, 0.8, 1)
2	(0.5, 0.3, 0.2)	(3, 1)	(0, 1)	(-3, 1)	(0.7, 0.8, 1)
3	(0.5, 0.3, 0.2)	(3, 1)	(0, 1)	(-3, 1)	(0.7, 1, 1.2)
**Parameter settings under H_a of heterogeneous covariate effects**					
**Scenario**	π	$\boldsymbol{\beta}_1$	$\boldsymbol{\beta}_2$	$\boldsymbol{\beta}_3$	σ
1	(0.5, 0.3, 0.2)	(5, 1.6)	(1, 1.5)	(-3, 1)	(0.7, 0.8, 1)
2	(0.5, 0.3, 0.2)	(3, 1.2)	(0, 1.1)	(-3, 1)	(0.7, 0.8, 1)
3	(0.5, 0.3, 0.2)	(3, 1.6)	(0, 1.5)	(-3, 1)	(1, 1.6, $\sqrt{2}$)

Table S13. True values of parameters in simulation study for sequential test, without equal variance assumption.

**Scenario**	π	$\boldsymbol{\beta}_1$	$\boldsymbol{\beta}_2$	$\boldsymbol{\beta}_3$	σ
1	(0.3, 0.5, 0.2)	(5, 0.3)	(1, 0.3)	(-3, 0.3)	(0.7, 0.8, 1)
2	(0.3, 0.5, 0.2)	(5, 0)	(1, 0.2)	(-3, 0.5)	(0.7, 0.8, 1)

Table S14. Type I error rate of overall test at $\alpha = 0.05$, percentage of correctly selected models, and mean of adjusted rand indices. Datasets were simulated under H_0 that x has no effect on the responses y (Table S10). The Naive I, II, III and WEST procedures were performed.

		n=500				n=300			
**Scenario**		**Naive I**	**Naive II**	**Naive III**	**WEST**	**Naive I**	**Naive II**	**Naive III**	**WEST**
1	Type I error rate	0.058	0.053	0.057	0.053	0.070	0.057	0.066	0.057
	% of correct models	0.940	0.945	0.940	0.945	0.924	0.937	0.924	0.937
	Mean ARI	1.000	1.000	0.999	1.000	0.995	0.999	0.994	0.999
2	Type I error rate	0.060	0.058	0.058	0.057	0.070	0.063	0.065	0.059
	% of correct models	0.934	0.936	0.934	0.937	0.913	0.916	0.913	0.919
	Mean ARI	0.995	0.996	0.995	0.996	0.992	0.992	0.991	0.992
3	Type I error rate	0.059	0.057	0.055	0.053	0.051	0.050	0.050	0.049
	% of correct models	0.020	0.020	0.024	0.020	0.015	0.015	0.015	0.016
	Mean ARI	0.726	0.727	0.726	0.727	0.727	0.727	0.727	0.727

Table S15. Power of overall test at $\alpha = 0.05$, percentage of correctly selected models, and mean of adjusted rand indices. Datasets were simulated under H_a that x has effect on the responses y (Table S11). The naive I, II, III and WEST procedures were performed.

		n=500				n=300			
**Scenario**		**Naive I**	**Naive II**	**Naive III**	**WEST**	**Naive I**	**Naive II**	**Naive III**	**WEST**
1	Power	1	1	1	1	1	1	1	1
	% of correct models	0.827	0.649	0.827	0.827	0.752	0.391	0.752	0.752
	Mean ARI	0.775	0.761	0.775	0.775	0.765	0.742	0.765	0.765
2	Power	1	1	1	1	1	1	1	1
	% of correct models	0.983	0.996	0.983	0.983	0.982	0.996	0.982	0.982
	Mean ARI	0.999	0.999	0.999	0.999	0.995	0.999	0.995	0.995
3	Power	0.803	0.801	0.799	0.796	0.578	0.577	0.578	0.568
	% of correct models	0.001	0.002	0.001	0.001	0.002	0.001	0.002	0.002
	Mean ARI	0.666	0.666	0.666	0.666	0.646	0.648	0.646	0.646

Table S16. Type I error rate of heterogeneous effect test at $\alpha = 0.05$, percentage of correctly selected models, and mean of adjusted rand indices. Datasets were simulated under H_0 that x has homogeneous effect on the responses y (Table S12). The Naive I, II, III and WEST procedures were performed.

		n=500				n=300			
**Scenario**		**Naive I**	**Naive II**	**Naive III**	**WEST**	**Naive I**	**Naive II**	**Naive III**	**WEST**
1	Type I error rate	0.055	0.054	0.053	0.050	0.076	0.068	0.074	0.062
	% of correct models	0.941	0.942	0.941	0.946	0.918	0.926	0.918	0.932
	Mean ARI	0.974	0.974	0.974	0.974	0.973	0.974	0.973	0.974
2	Type I error rate	0.073	0.065	0.069	0.062	0.118	0.100	0.108	0.070
	% of correct models	0.910	0.918	0.910	0.921	0.738	0.717	0.739	0.766
	Mean ARI	0.883	0.884	0.882	0.883	0.833	0.833	0.829	0.834
3	Type I error rate	0.104	0.074	0.097	0.045	0.159	0.118	0.154	0.067
	% of correct models	0.509	0.539	0.516	0.536	0.283	0.323	0.288	0.321
	Mean ARI	0.729	0.733	0.730	0.733	0.684	0.692	0.685	0.692

Table S17. Power of heterogeneous effect test at $\alpha = 0.05$, percentage of correctly selected models, and mean of adjusted rand indices. Datasets were simulated under H_a that x has heterogeneous effect on the responses y (Table S12). The Naive I, II, III and WEST procedures were performed.

		n=500				n=300			
**Scenario**		**Naive I**	**Naive II**	**Naive III**	**WEST**	**Naive I**	**Naive II**	**Naive III**	**WEST**
1	Power	0.953	0.954	0.959	0.957	0.780	0.771	0.774	0.755
	% of correct models	0.962	0.962	0.953	0.951	0.734	0.754	0.721	0.698
	Mean ARI	0.924	0.924	0.924	0.924	0.906	0.910	0.903	0.903
2	Power	0.360	0.334	0.342	0.311	0.330	0.295	0.321	0.282
	% of correct models	0.093	0.169	0.054	0.054	0.035	0.096	0.018	0.015
	Mean ARI	0.673	0.689	0.667	0.672	0.631	0.647	0.628	0.635
3	Power	0.785	0.781	0.785	0.777	0.603	0.598	0.603	0.587
	% of correct models	0.023	0.047	0.022	0.021	0.009	0.029	0.008	0.007
	Mean ARI	0.544	0.544	0.544	0.544	0.536	0.537	0.536	0.535

Table S18. The results of sequential test at $\alpha = 0.05$. Datasets are simulated under the settings in Table S13 The Naive I, II, III and WEST procedures were performed.

		n=500				n=300			
**Scenario**		**Naive I**	**Naive II**	**Naive III**	**WEST**	**Naive I**	**Naive II**	**Naive III**	**WEST**
1	Power (Overall test)	1.000	1.000	1.000	1.000	0.999	0.999	0.999	0.999
	Type I error rate (Heterogeneous test)	0.065	0.062	0.065	0.060	0.061	0.057	0.061	0.056
	% of correct models	0.933	0.935	0.933	0.936	0.938	0.941	0.938	0.943
	Mean ARI	0.973	0.973	0.973	0.973	0.972	0.972	0.972	0.972
2	Power (Overall test)	0.996	0.996	0.996	0.996	0.944	0.944	0.937	0.939
	Power (Heterogeneous test)	0.968	0.968	0.968	0.967	0.832	0.832	0.826	0.830
	% of correct models	0.966	0.967	0.966	0.966	0.827	0.832	0.819	0.823
	Mean ARI	0.963	0.963	0.963	0.963	0.959	0.960	0.960	0.960

4. Additional information in real data analysis

4.1. Application to Chiroptera data

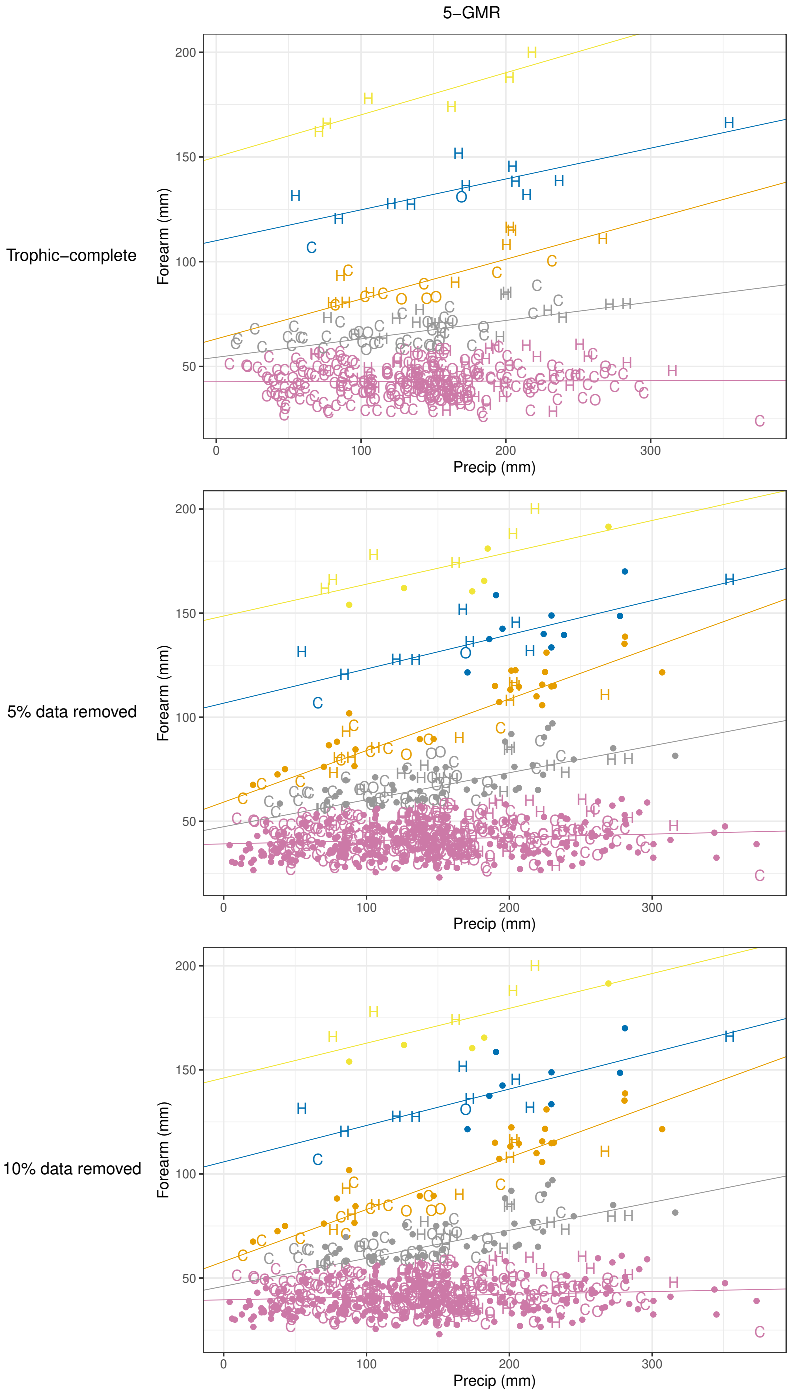

Figure S1. Scatterplot of Chiroptera data clustered by the fitted groups by 5-GMR model with the pre-classified trophic level, in trophic-complete, 5% and 10% randomly removed data, where colors refer to the fitted groups and the letters in plot refer to the pre-classified trophic level,.

4.2. Application to diabetes data

4.2.1. Data description

We apply our proposed testing procedures to the data obtained from the diabetes study of Reaven [**?**]. In this study, 145 nonobese individuals participated. During the first three days, all individuals ate the same formula diet and their steady state plasma glucose (sspg) levels were measured. After that, they were given an oral glucose. The area under plasma insulin curve (insulin) and the area under plasm glucose curve (glucose) were recorded during the following three hours. Reaven suggests a linear relationship between the area under plasma glucose curve and the sspg level, but there is no linear trend between the area under plasma insulin curve and the sspg [**?**]. Motivated by this reason, Scrucca et al. proposed to use multivariate Gaussian mixture (MGM) model to cluster these subjects [**?**]. While MGM aims to cluster individuals based on the joint distribution of these variables, GMR models attempt to model the effect of sspg on the area under plasma insulin curve. Reaven also suggests dividing the participants into three groups based on their diabetes status: overt diabetes (plasma glucose levels higher than ll0mg/dl), chemical diabetes (plasma glucose levels beyond 185 mg/dl and 140 mg/dl at one and two hours after the glucose load), and normal diabetes [**?**]. This conventional classification information will not be used in our clustering analysis but will be used as a reference to compare the results from our clustering analysis. The data set is also available in the R software [**?**] package "mclust" [**?**].

4.2.2. Model fitting and hypothesis tests results

We fit the data using the GM, Homo-GMR, and GMR models. The order of these three mixture models is unknown. Thus, we consider a range of choices for G from 2 to 5. P-values of the overall test and the heterogeneous effect test of sspg effects on insulin are reported in Table S19. For all proposed testing procedures, the p-values for the overall test are all very small such that the null hypothesis that the covariate sspg has no effect on the response insulin is rejected. The p-values for the heterogeneous effect test are also all very small such that the null hypothesis that the covariate has homogeneous effects on the response across all components is rejected. When performing the sequential test, because the null hypothesis of the overall test is rejected, step 2 for testing the heterogeneity of covariate effects is performed and gives the same result as in the heterogeneous effect test, therefore they are not reported here. We can conclude that there is significant evidence to indicate that the ability of a person to absorb glucose for the same glucose loads (sspg) affects the three hour oral glucose tolerance (insulin) differently. Furthermore, based on the test results, the 3-component GMR model is suggested as the best-fitting model.

Table S19. P-values associated with the overall and heterogeneous effect tests for sspg effects, and the optimal model selected in sequential test, based on the four testing procedures

**Procedure**	**Overall test**	**Heterogeneity effects test**	**Best model by Sequential test**
Naive I	$2.589 * 10^{-16}$	$8.419 * 10^{-16}$	3-GMR
Naive II	$2.589 * 10^{-16}$	$8.419 * 10^{-16}$	3-GMR
Naive III	$2.589 * 10^{-16}$	$8.419 * 10^{-16}$	3-GMR
WEST	$1.053 * 10^{-15}$	$2.278 * 10^{-13}$	3-GMR

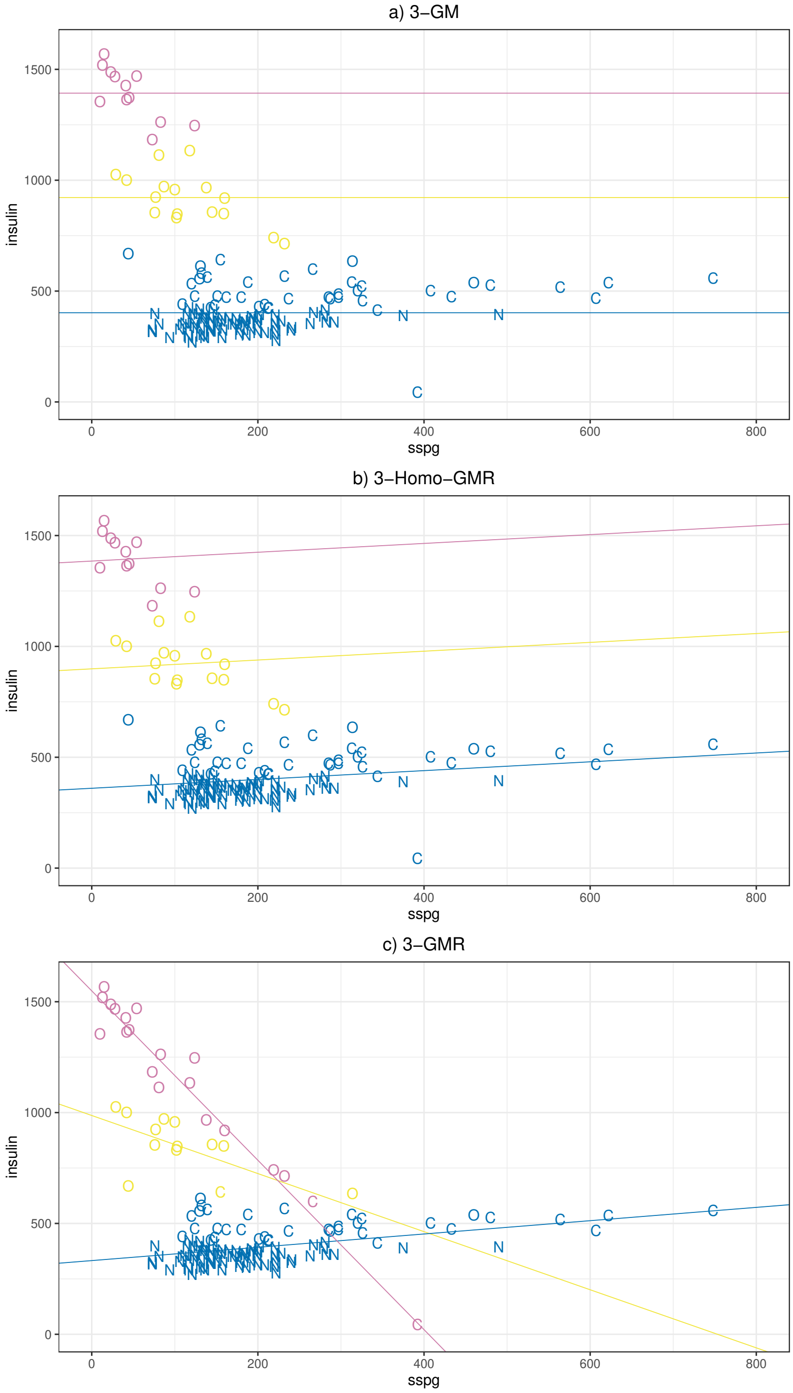

Figure S2. Scatterplots of diabetes data clustered by the fitted groups and classified groups via 3-GM, 3-Homo-GMR and 3-GMR model, respectively, where colors refer to the fitted groups and the letters in plot refer to the classified groups: O (overt diabetes), C (chemical diabetes), and N (normal).

Table S20. Estimated parameters of 3-GMR model for the diabetes data.

**Parameter**	**3-GMR**
β_{0g}	(1548.96, 986.86, 332.42)
Standard error	(67.66, 266.55, 16.17)
β_{1g}	(-3.82, -1.31, 0.30)
Standard error	(0.45, 1.33, 0.05)
π_g	(0.14, 0.11, 0.75)
Standard error	(0.05, 0.04, 0.05)
σ_g	75.77
Standard error	12.20

The scatterplots of sspg versus insulin fitted by three different mixture models are displayed in Figure S2, with the colors representing the different clusters fitted. The diabetes status of each observation is indicated by letters "O", "C", and "N" for overt diabetes, chemical diabetes, and normal respectively. Figure S2c) represents the clustering result of the GMR models with sspg having different effects on the insulin area for different clusters. Under 3-component GMR model, the sspg has a positive effect on the insulin in the blue cluster where sspg has different levels of negative effects on the insulin in the pink and yellow clusters. Parameter estimates of mixing proportions π's, regression intercepts and coefficients $\boldsymbol{\beta}$'s, and standard deviation of the optimal models following the WEST procedure are reported in Table S20.

Interestingly, under the 3-component GMR model, subjects clustered into components 1 and 2 are all classified as overt diabetes except three subjects are classified as chemical diabetes, and they all response negatively to the increased sspg levels but with different levels as -3.81 for cluster 1 and -1.31 for cluster 2. Subjects in component 3 are mostly normal or classified as chemical diabetes and their insulin levels respond positively with the increased sspg. The 3-component GMR model is suggested to be preferable to the 2-component GMR model by their BIC values. It might be worth further investigating, what are the additional factors that influence the insulin response among overt diabetes subjects such that subjects with overt diabetes respond to sspg differently in their insulin levels.

5. Source code

We used R to implement all the methods. The source code, including functions for EM algorithm and test procedures, is available at `https://github.com/c2gan/GMR-JAS.git`.
